# HTRF-based assay for detection of mono-ADP-ribosyl hydrolyzing macrodomains and inhibitor screening

**DOI:** 10.1016/j.isci.2024.110333

**Published:** 2024-06-20

**Authors:** Niklas Ildefeld, Dieter Steinhilber, Ewgenij Proschak, Jan Heering

**Affiliations:** 1 Institute of Pharmaceutical Chemistry, Goethe-University of Frankfurt, Biocenter, Max-von-Laue-Str. 9, 60438 Frankfurt/Main, Germany; 2 Fraunhofer Institute for Translational Medicine and Pharmacology ITMP, Theodor-Stern-Kai 7, 60596 Frankfurt/Main, Germany; 3 Fraunhofer Cluster of Excellence Immune-Mediated Diseases CIMD, Theodor-Stern-Kai 7, 60596 Frankfurt/Main, Germany

**Keywords:** Pharmacology, Biochemistry, Microbiology, Cell biology

## Abstract

The COVID-19 pandemic has highlighted the lack of effective, ready-to-use antivirals for the treatment of viruses with pandemic potential. The development of a diverse drug portfolio is therefore crucial for pandemic preparedness. Viral macrodomains are attractive therapeutic targets as they are suggested to play an important role in evading the innate host immune response, making them critical for viral pathogenesis. Macrodomains function as erasers of mono-ADP-ribosylation (deMARylation), a post-translational modification that is involved in interferon signaling. Herein, we report the development of a modular HTRF-based assay, that can be used to screen for inhibitors of various viral and human macrodomains. We characterized the five most promising small molecule SARS-CoV-2 Mac1 inhibitors recently reported in the literature for potency and selectivity and conducted a pilot screen demonstrating HTS suitability. The ability to directly detect enzymatic activity makes the DeMAR assay a valuable addition to the existing tools for macrodomain drug discovery.

## Highlights

• HTRF-based assay system detecting enzymatic removal of mono-ADP-ribosylation
• Modular system allows rapid adaptation for new mono-ADPr hydrolyzing macrodomains
• HTS-suitability demonstrated with SARS-CoV2 Mac1
• Wash protocol enables study of nucleotide-like macrodomain inhibitors

## Graphical abstract

**Figure:**
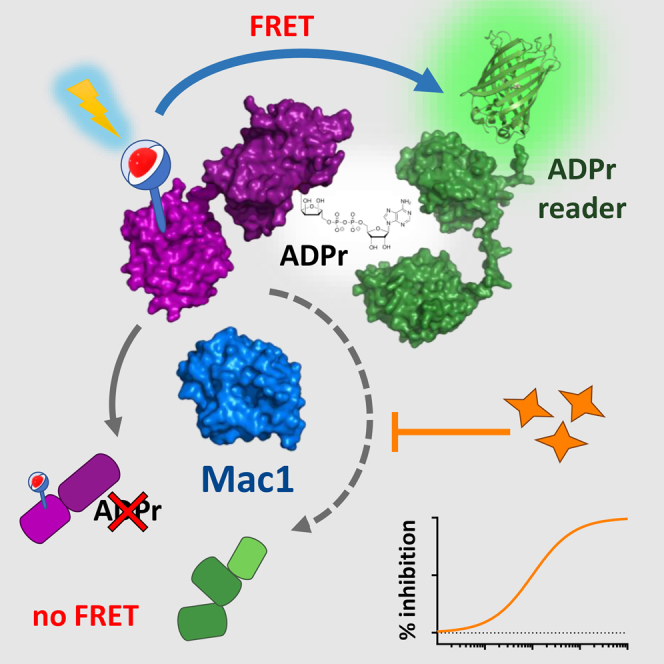

## Introduction

The World Health Organization announced on June 5th, 2023, that, after three and a half years, COVID-19, the disease caused by Severe Acute Respiratory Syndrome Coronavirus-2 (SARS-CoV-2), is no longer a public health emergency of international concern (WHO). Acquired immunity through vaccination and infection, as well as the onset of variants with lower pathogenicity have resulted in a significant reduction in infection fatality rates. However, COVID-19 remains a major health threat for vulnerable populations such as the elderly and immunocompromised individuals that are at risk for a severe course of disease.^1^ Moreover, many patients experience ongoing complaints after SARS-CoV-2 infection that cannot be adequately treated,^2^ and mutational escape is still a matter of concern that needs to be addressed.^3^ Beyond that, the outbreak has highlighted the lack of effective antivirals available to target a broad range of viruses and to counter future zoonotic outbreaks. In order to meet these challenges, and for epidemic preparedness, the validation of novel drug targets to expand the pool of antiviral therapies is of great importance.

Currently, the 3C-like protease inhibitor nirmatrelvir, and the RNA polymerase inhibitors molnupiravir and remdesivir are the only approved antivirals that directly target SARS-CoV-2. Inhibition of the conserved macrodomain-1 (Mac1) is expected to complement these mechanisms by affecting both viral replication and the innate interferon response.^4^^,^^5^

Mac1 belongs to the larger family of MacroD-type macrodomains, which have a highly conserved three-layered α/β/α fold. It is part of the large non-structural protein 3 (nsp3), and present in all coronaviruses. Macrodomains have the ability to modulate ADP-ribosylation either by binding to ADP-ribose (reader) or by hydrolyzing ADP-ribose from target substrates (eraser).^6^^,^^7^^,^^8^^,^^9^ ADP-ribosylation is a reversible and dynamic post-translational modification (PTM) catalyzed by ADP-ribosyl transferases (ARTs, also known as PARPs or ARTDs) that transfer an ADP-ribose moiety from NAD^+^ to target proteins or nucleic acids. ADP-ribose (ADPr) can either be transferred as mono-ADP-ribose (MAR), or successive units of ADP-ribose can be covalently linked through glycosidic bonds to preceding units to form a branched or linear chain of poly-ADP-ribose (PAR).^7^^,^^10^ While ADP-ribosylation has been implicated in diverse cellular processes such as DNA damage response, RNA metabolism or stress granule formation, in particular MARylation has recently emerged as a critical modulator of the interferon (IFN) response, both upstream and downstream of the signaling cascade, suggesting that ADP-ribosylation plays an important role in the host innate immune response to viral infection.^4^^,^^11^^,^^12^

Mac1 is thought to counteract PARP-mediated antiviral ADP-ribosylation through its deMARylating activity, making it a major mechanism by which the virus evades the innate immune response, as shown for SARS-CoV-2 by two recent publications.^13^^,^^14^ The reported data suggest that there are strong differences in how different coronaviruses require Mac1 in terms of viral replication, but it is consistent that viruses containing a complete Mac1 deletion or a catalytically inactive Mac1 tend to induce an elevated IFN response particularly *in vivo*, have reduced viral loads, and do not cause significant disease.^13^^,^^14^

This is of particular interest since Mac1 is structurally highly conserved not only in coronaviruses, but also in alphaviruses, making adaptation of a potential therapeutic concept a conceivable option.^5^^,^^11^^,^^15^

Indications for viral macrodomains counteracting specific PARPs is strongest for PARP14, which is involved in DNA replication stress management, transcription, and control of immunity.^16^ PARP14 enhances the host type I interferon (IFN-I) response to lipopolysaccharide (LPS) and viral infection. In three human lung cell lines expression of PARP14 was shown to be upregulated following infection with SARS-CoV-2.^17^ Furthermore, PARP14 also induces IFN-I upon treatment with poly(I:C), a double-stranded RNA mimic, and A549 cells with a deletion of PARP14 were unable to mount a full IFN-I response after infection with MHV coronavirus (mouse hepatitis virus).^4^ PARP14 has both ADP-ribosyl transferase (ART catalytic domain) and hydrolase (macrodomain 1) activities, both interestingly restricted to MAR, and auto-MARylation of the PARP14 catalytic domain was reported to be efficiently reversed by SARS-CoV-2 Mac1 *in vitro* and in cells.^18^^,^^19^ This suggests that PARP14 is directly involved in the establishment of the innate immune response in CoV-infected cells and that viral macrodomains with MAR hydrolase activity are the product of an evolutionary arms race to counteract the antiviral response mediated by PARP14 and other PARPs.

Since viral macrodomains appear to be critical virulence factors, numerous research efforts have been undertaken to identify potent SARS-CoV-2 Mac1 inhibitors. Therefore, in addition to crystallographic and virtual screening approaches, several protein-based *in vitro* assay systems have been established that are suitable for high-throughput screening. In most cases, these assays detect binding to Mac1, which is detected as displacement of tracer molecules, which are pseudo-substrates that cannot be hydrolyzed. Consequently, observed binding of compounds does not necessarily imply that identified compounds also inhibit its enzymatic activity.^20^^,^^21^^,^^22^^,^^23^

For example, the assays of Schuller et al.^24^ and Roy et al.^22^ utilize synthetic MARylated peptides as tracers which cannot be hydrolyzed by Mac1 due to mono-ADP-ribose not being linked to a Glu/Asp residue but instead to a Lys sidechain. On the other hand, the fluorescence polarization assay developed by Anmangandla et al.^23^ employs TAMRA-ADPr as tracer. However, there is also a luminescence-based ADP-ribosylhydrolase assay (ADPr-Glo) that was reported by Dasovich et al.^25^ This assay utilizes an MARylated fusion protein that is a suitable substrate for Mac1 resulting in the release of free ADP-ribose. Free but not terminal ADP-ribose is then further hydrolyzed to AMP by the phosphodiesterase NudF in order to finally detect generated AMP with the commercially available AMP-Glo kit.

The ADPr-Glo assay is well suited for identification of Mac1 inhibitors, but the fact that its readout strategy relies on several additional enzymes (NudF, a kinase, and a luciferase) could pose the risk of identifying false positives due to inhibition of one of these enzymes by a test compound. However, this can be countered by simply retesting the hits in a control experiment without Mac1 but in the presence of free ADPr.

Despite these different approaches, SARS-CoV-2 Mac1 has proven to be a challenging target for drug discovery, and biochemical screening so far had limited success in identifying potent nanomolar Mac1 inhibitors. Nevertheless, Gahbauer et al.^26^ recently reported for the first time the structure-based discovery of several different compounds with low to sub-micromolar affinity for Mac1, which were generated by fragment merging, thereby demonstrating that IC_50_ values below 1 μM are achievable at least in the assay of Schuller et al.^24^

Herein, we report the development of an enzymatic assay with indirect readout that complements recent research tools for the identification of viral macrodomain inhibitors. The assay, which is based on homogeneous time-resolved fluorescence energy transfer (HTRF), utilizes the specific interaction between MARylated PARP10cat (ARTD10) and PARP14 (ARTD8) Mac2/3, which is well described in the literature.^27^^,^^28^ PARP10 belongs to the class 2 of ARTD family proteins which are limited to mono-ADP-ribosylation as they use substrate-assisted catalysis to transfer a single ADP-ribose unit onto a substrate.^29^ PARP10cat is capable of intermolecular auto-MARylation,^29^^,^^30^ and in cell culture it was shown that MARylation by endogenous PARP10 modulates the activity of e.g., glycogen synthase kinase 3β.^31^ De-MARylation of PARP10cat by SARS-CoV2 Mac1 was previously demonstrated using PARP10cat that had been auto-MARylated *in vitro* with biotin-NAD^+^, and after incubation with Mac1, the biotin was detected on western blot.^32^ PARP14 has three macrodomains of which Mac2&3 directly bind MARylated PARP10cat, whereas PARP14 Mac1 has a 100-fold lower affinity for ADPr compared to PARP14 Mac3.^27^^,^^28^ The function of PARP14 Mac1 has long remained elusive, and only recently has this macrodomain been reported to have ADP-ribosyl hydrolase activity.^18^^,^^19^ Several MAR detection reagents based on PARP14 Mac2/3 have been described in the literature. For example, an antibody-like detection reagent was generated by fusing PARP14 Mac2/3 to the constant fragment (Fc) of rabbit IgG.^33^ It is used for detection on western blot and was shown to exclusively detect MARylation. Furthermore, it was reported to detect MARylated PARP10cat with good sensitivity as well as high specificity versus unmodified PARP10cat.^34^

In our HTRF-based deMARylation assay (DeMAR assay), SNAP-tagged PARP10cat is coupled to terbium-cryptate (Tb), which acts as the FRET donor, whereas PARP14 Mac2/3 is expressed as a fusion protein with sGFP, which is the corresponding FRET acceptor. The recruitment of PARP14 Mac2/3 by MARylated PARP10cat generates a high and robust HTRF signal. Consequently, the addition of a viral macrodomain prevents complex formation due to its deMARylating activity, resulting in a decay in HTRF. This allows compounds to be tested for their ability to inhibit Mac1 activity, which is indicated by a preservation of the HTRF signal (signal on). In 384-well format, we first characterized eight different macrodomains (both viral as well as host factors) for their deMARylating activity on PARP10cat and then validated the five most promising small molecule SARS-CoV-2 Mac1 inhibitors recently reported in the literature for potency and selectivity. In addition, we performed a pilot screen on SARS-CoV-2 Mac1 of 2528 compounds using the add-only protocol of the assay to demonstrate that the DeMAR assay is well suited for high-throughput screening.

## Results

**Figure 1:**
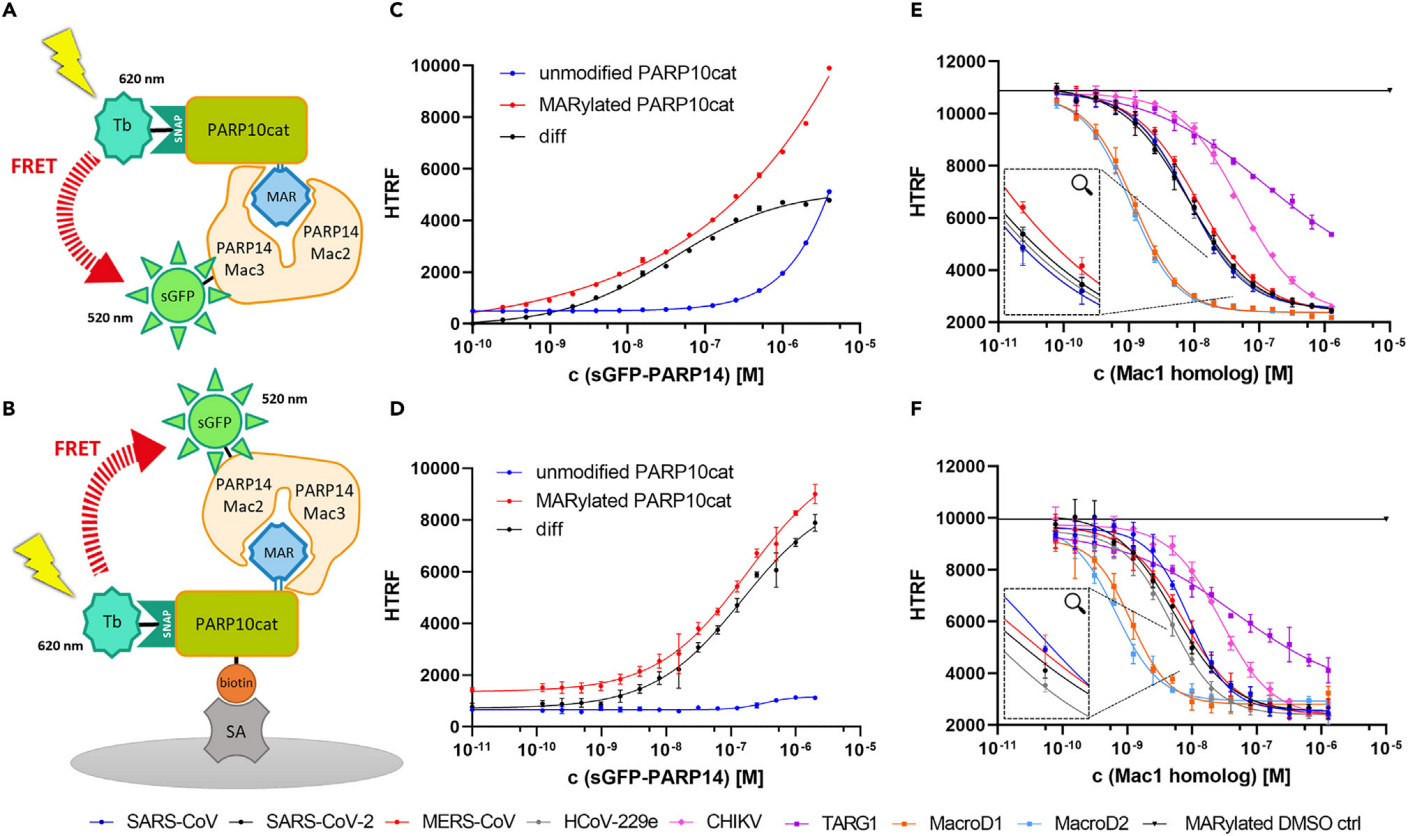
Evaluation of the readout system and the activity of eight Mac1 homologs in one-pot (top) and wash setup (below) (A and B) Schematic representation of the MAR-PARP10cat and PARP14 Mac2/3 FRET pair formation in the one-pot setup (top) and the wash setup (below). (C and D) Reader sGFP-PARP14 Mac2/3 was titrated from 0.2 nM up to 4 μM against either 6 nM of MARylated (red) or unmodified (blue) Tb-labeled PARP10cat. The specific binding curve (black) was determined by subtracting the unmodified curve (unspecific binding) from the MARylated curve (overall binding). A DMSO control is the lowest concentration in each dataset. Data represent the means ± SD of 3 technical replicates for each construct. R^2^ for each curve equals >99%. (E and F) Each Mac1 homolog construct was titrated from 0.08 nM up to a concentration of 1.28 μM against 3 nM (one-pot setup) or 6 nM (wash setup) MARylated Tb-PARP10cat. The readout was performed with 200 nM sGFP-PARP14 Mac2/3 as described in STAR Methods. A negative control (no Mac1) was included in each experiment. Data represent the means ± SD of 3 technical replicates for each Mac1 homolog. R^2^ for each curve in the one-pot setup equals >99%, in the wash setup it equals >97%. SARS-CoV (*dark blue*), SARS-CoV-2 (*black*), MERS-CoV (*red*), HCoV-229e (*gray*), CHIKV (*pink*), TARG1 (*purple*), MacroD1 (*orange*), MacroD2 (*light blue*).

The first step in the development of the HTRF-based deMARylation assay was to evaluate the readout system based on the specific interaction of the “reader macrodomains” PARP14 Mac2/3 with the MARylated catalytic domain of PARP10.^27^^,^^28^^,^^29^ We demonstrated that only the auto-MARylated but not the unmodified PARP10cat is able to recruit PARP14 Mac2/3. Therefore, we titrated sGFP-PARP14 (from 0.2 nM up to 4 μM) against either MARylated or unmodified Tb-PARP10cat, each at a fixed concentration of 6 nM (Figure 1C). At up to 0.1 μM sGFP-PARP14 a concentration-dependent increase in HTRF was observed only with MARylated PARP10cat, indicating that, PARP14 Mac2/3 is only recruited by MARylated PARP10cat and does not possess hydrolytic activity. The steep increase in HTRF in the unmodified PARP10cat titration occurring at concentrations higher than 1 μM sGFP-PARP14 is presumably explainable by the effect of diffusion enhanced FRET. Consequently, we chose a concentration of 200 nM sGFP-PARP14 for the deMARylation assay to obtain a most sensitive signal that results only from the specific interaction between MARylated PARP10cat and PARP14 Mac2/3. In addition, titration experiments were performed with different fixed Tb-PARP10cat concentrations to determine the ideal FRET donor concentration (Figure S1). Since reduction of Tb-PARP10cat concentration to 3 nM resulted in only a small loss of signal, we decided on using this concentration of Tb- PARP10cat to reduce costs. The influence of glycerol and DMSO content was also evaluated. 5% (w/v) glycerol only marginally reduced maximal HTRF signal, slightly reduced %CV compared to the experiment with 1% glycerol, and resulted in a Hill slope of approximately −1. Variations in the DMSO content between 0.5 and 3.0% did not affect assay performance with SARS-CoV-2 (Figures S2A and S2B).

Once these parameters were set, we examined eight different Mac-1 homologs for their hydrolytic activity on MARylated PARP10cat. In addition to the SARS-CoV-2 Mac1, we also selected the macrodomains of the coronaviruses SARS-CoV, MERS-CoV, and HCoV-229e, as well as the macrodomain of the Chikungunya (CHIKV) alphavirus. Moreover, the human macrodomains TARG1, MacroD1 and MacroD2 were included in the set as obvious off-targets. Each Mac1 homolog was titrated from 0.08 nM up to 1.28 μM against 3 nM Tb-PARP10cat, and after incubation for 1 h, the readout was performed by addition of 200 nM sGFP-PARP14 (Figure 1E). As expected, increasing macrodomain concentrations led to a decrease in HTRF in all experiments, indicating that all tested macrodomains are enzymatically active and able to remove mono-ADPr from PARP10cat. Moreover, except for TARG1, the same lower HTRF plateau was reached in all separate experiments, suggesting a complete removal of MARylation from PARP10cat. TARG1 represents a special case, which is not entirely unexpected since it has been described to have a different catalytic mode compared to the other human macrodomains MacroD1 and D2, and to also possess the unique ability to accept as substrate both MARylated and PARylated side chains of aspartate and glutamate residues.^9^^,^^35^ As further proof of concept, we also tested a mutant SARS-CoV-2 Mac1 in which the ADPr binding pocket is sterically blocked (G131V). As expected, the mutant macrodomain was inactive (Figure S3). A time course experiment was also performed with SARS-CoV-2 Mac1 wt, showing that 1 h was sufficient to deMARylate >80% of the substrate, depending on the amount of Mac1 added; Figure S4A.

For the deMARylation assay, in order to test potential inhibitors with high sensitivity, the macrodomain concentration was adjusted in a way that approximately 80–90% of the MARylated PARP10cat was processed within 1 h, respectively. Since the tested macrodomains have different deMARylation activities (Table 1), the chosen macrodomain concentrations, hence, vary to obtain a similar assay window for each macrodomain. In case of the coronavirus macrodomains SARS-CoV, SARS-CoV-2, MERS-CoV and HCoV-229e, the concentration was set at 80 nM, while the human macrodomains MacroD1 and MacroD2 were applied at 10 nM; the concentration used for CHIKV is 300 nM and for TARG1 2.5 μM.

**Table 1:** Macrodomain activities

EC_80_ [nM]	SARS-CoV	SARS-CoV-2	MERS-CoV	HCoV-229e	CHIKV	TARG1	MacroD1	MacroD2
**One-pot (1 h)**	34	41	53	36	218	∼2256	4	3
**Wash (2 h)**	25	22	29	15	100	∼ 830	3	2

**Figure 2:**
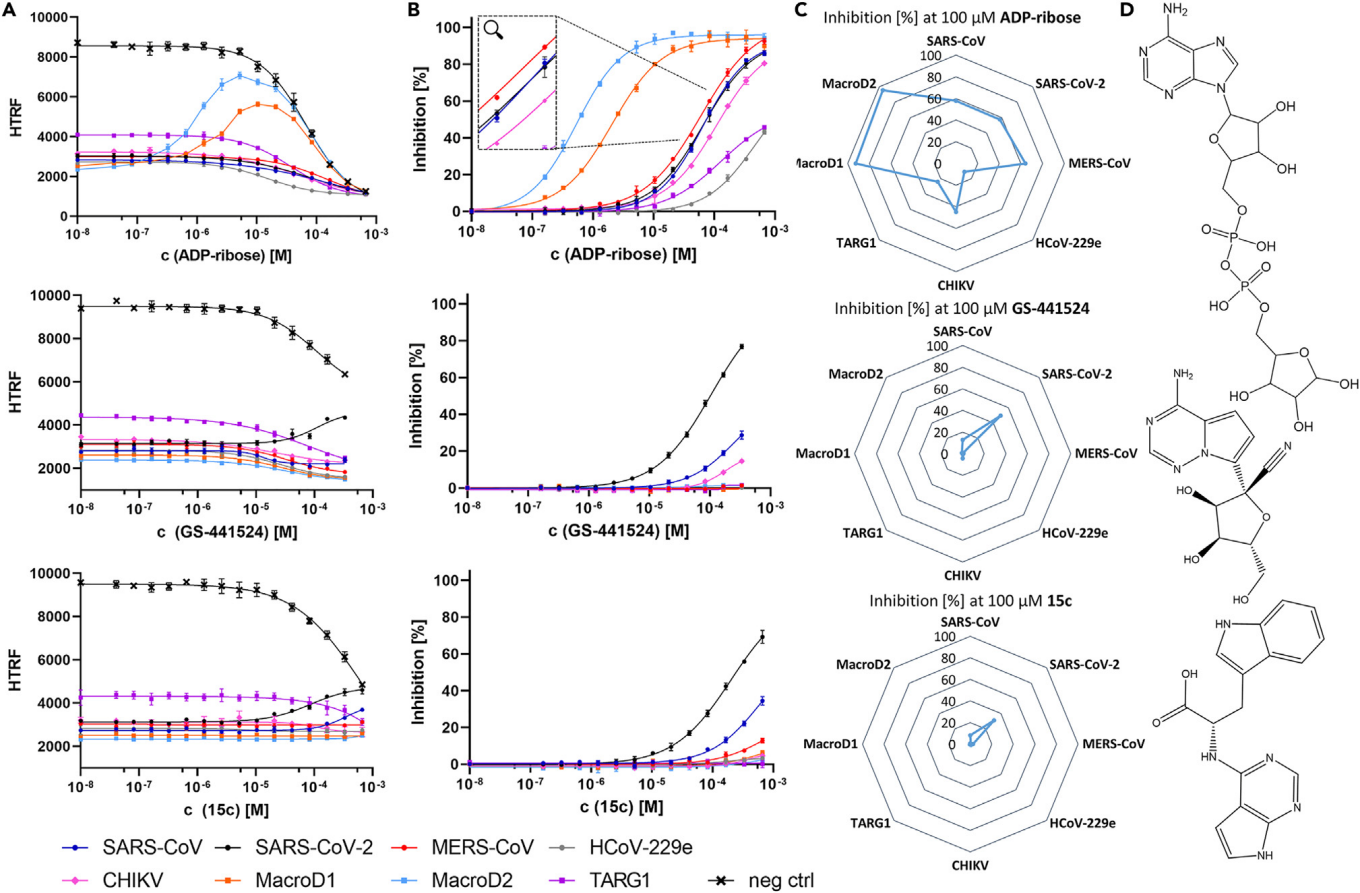
Examination of the inhibitory potential of ADP-ribose, GS-441524, and 15c on eight different Mac1 homologs (A) One-pot setup: Each compound was titrated up to 667 μM to 3 nM Tb-PARP10cat, and a defined Mac1 concentration; for SARS-CoV (dark blue), SARS-CoV-2 (black), MERS CoV (red), HCoV-229e (gray) 80nM, for CHIKV (pink) 300 nM, for TARG1 (purple) 2.5 μM, and for MacroD1 (orange), MacroD2 (light blue) 10 nM. The readout was performed with 200 nM sGFP-PARP14 Mac2/3 as described in STAR Methods. A negative control without Mac1 homolog addition was included in each experiment. A DMSO control is the lowest concentration in each dataset. Data represent the means ± SD of 4 technical replicates for each construct. R^2^ for each curve equals >99%. For MacroD1 and D2 tested with ADP-ribose, GraphPad Prism 9 could not generate fitting curves; connecting lines are shown instead. (B) Wash setup: Each compound was titrated up to 667 μM to 6 nM immobilized Tb-PARP10cat, and a defined Mac1 concentration (as described in (A)). The readout was performed with 200 nM sGFP-PARP14 Mac2/3 after washing as described in STAR Methods. Curves were fitted based on the HTRF signals and then normalized according to the respective upper and lower plateau. A DMSO control is the lowest concentration in each dataset. Data represent the means ± SD of 3 technical replicates for each construct. R^2^ for each curve equals >99%. (C) Radar plots were generated based on the data from the wash setup experiments to visualize the inhibition [%] at 100 μM of each compound tested for all eight macrodomains examined. (D) Structural formulas of the tested compounds; structures were created using ChemDraw.

With all parameters defined, we first tested free ADPr, as the most studied ligand, for its inhibitory potential against the recombinant macrodomains to demonstrate that the deMARylation assay is suitable for identifying viral macrodomain inhibitors. For this purpose, ADPr was titrated from a concentration of 40 nM up to 667 μM, and after 1 h of incubation with the fixed PARP10cat and Mac1 homolog, the readout was performed (Figure 2A). Here we faced a conceptual limitation of the assay setup. The readout system relies on the recognition of monomeric ADPr bound to PARP10cat by PARP14 Mac2/3. In a one-pot setup, free ADPr does not only inhibit the macrodomain of interest, but also blocks the binding sites on PARP14 Mac2/3, resulting in a quenching of the readout. This effect can be observed in the negative control, which shows a distinct sigmoid shape, allowing the determination of an IC_50_ value of 67 μM of ADPr against PARP14 Mac2/3 (Figure 2A). Furthermore, also in all separate experiments with macrodomains from about 5 μM ADPr onwards the HTRF signal trended to the same lower plateau as observed in the negative control, implying that above 5 μM ADPr the quenching effect (vide supra) overrides any observable inhibitor effect. Interestingly, in the experiments with the human macrodomains MacroD1 and D2, the HTRF signal was at least partially restored below 5 μM ADPr, indicating that particularly these two human macrodomains have a high affinity for free ADPr, which is consistent with the literature.^15^^,^^23^^,^^36^ The ADPr titration experiments demonstrate that the designed assay is in principle a suitable tool for identifying macrodomain inhibitors, with the limitation that in this setup inhibitors must be selective for the investigated macrodomain with low affinity for PARP14 Mac2/3. As PARP14 has been described to play a crucial role in the regulation of the innate response,^4^^,^^8^ this limitation can also be seen as a direct off-target test and can be considered an interesting feature of the assay.

However, in order to discriminate a wide range of compounds based on their inhibitory effects against different macrodomains, we decided to adapt the deMARylation assay by adding a wash step prior to the addition of sGFP-PARP14. In this way, signal readout suppression due to inhibition of PARP14 Mac2/3 and possible FRET donor quenching should be prevented, as the macrodomain, cleaved ADP-ribose and tested compounds are removed from the system before readout. We expressed and purified a biotin-labeled PARP10cat construct (Avi-SNAP-PARP10cat) that allows for the immobilization of PARP10cat on streptavidin-coated microtiter plates (Figure 1B). In this wash setup, twice the concentration of MARylated and biotin-labeled PARP10cat is used for coupling compared to the one-pot setup in order to obtain comparable FRET signals in both setups. In accordance with the evaluation of the readout system for the one-pot setup, titration experiments with sGFP-PARP14 (from 0.1 nM up to 2 μM) were carried out in the wash setup. The results indicate that sGFP-PARP14 Mac2/3 is consistently recruited in both the one-pot and the wash setup (Figure 1D). At a concentration of 200 nM sGFP-PARP14 the signal windows were comparable in both setups, and hence, this concentration was also chosen for the wash protocol. The previously studied macrodomains were analogously tested for their deMARylating activity (Figure 1F). The observed deMARylation was slightly slower compared to the one-pot protocol. This can be explained by the fact that the MARylated substrate is now immobilized instead of freely diffusing. However, with an extended Mac1 incubation time of 2 h instead of 1 h, about 80% of the substrate was again deMARylated at the same macrodomain concentrations as used in the final one-pot protocol, allowing for the best comparability between the two setups (Table 1; Figure S4B).

Once the wash setup was established, we again demonstrated the suitability of the setup by testing the inhibitory potential of free ADPr in a titration experiment (Figure 2B). As in the one-pot setup, ADPr was titrated to a concentration of 667 μM against immobilized PARP10cat and the selected Mac1 homolog. After 2 h of incubation, the described wash step was performed before adding sGFP-PARP14 for detection. As expected, no readout quenching was observed and instead with all tested macrodomains the HTRF signals are recovered to some extent in a concentration-dependent manner. As already suspected from the one-pot experiment, free ADPr inhibits the two human macrodomains MacroD1 and MacroD2 with particularly high potency (IC_50_: 2 μM and 0.5 μM, respectively). This is in agreement with their reported high affinities for free ADPr (*K*_d_: 0.9 μM and 0.15 μM, respectively).^36^ The other human macrodomain TARG1 is apparently not potently inhibited by free ADPr, which is consistent with the observation that the TARG1 dose-response curves in the Mac1 activity experiments (Figures 1E and 1F) have a different Hill slope compared to the other macrodomains (approximately −0.5 compared to −1.0). The macrodomains of the beta-coronaviruses SARS-CoV, SARS-CoV-2 and MERS-CoV are substantially less affected, with the potency of ADPr being approximately 10-times lower compared to MacroD1 (IC_50_: 65 μM, 66 μM and 60 μM, respectively). Interestingly, the macrodomain of alphacoronavirus HCoV-229e shows a notably weaker affinity for free ADPr (IC_50_: ∼460 μM), although all coronavirus macrodomains cluster close to one another in the Mac1 activity experiments (Figures 1E and 1F). Moreover, free ADPr inhibits the CHIKV macrodomain with approximately 2-fold lower potency compared to that observed on the beta-coronavirus macrodomains (IC_50_: 110 μM).

After demonstrating the effect of free ADPr, we tested several SARS-CoV-2 Mac1 inhibitors described in recent literature on our panel of eight macrodomains in both the one-pot and wash-step deMARylation assay. First, we evaluated the nucleotide-based GS-441524, the active metabolite of remdesivir, which is reported to bind to SARS-CoV-2 Mac1 with a K_d_ of 10 μM^23^^,^^37^^,^^38^ (Figure 2). In addition, GS-441524 has been shown to reduce the activity rate of SARS-CoV-2 Mac1 in a gel-based assay.^38^ In fact, in the one-pot setup, GS-441524 slightly inhibits SARS-CoV-2 Mac1, while all other macrodomains are unaffected (Figure 2A). However, the significance of this experiment is limited, since GS-441524 causes a clear and concentration-dependent loss of signal in the counter-screen, implying that GS-441524 also targets PARP14 Mac2/3. On the contrary, the inhibitory activity of GS-441524 can be well examined in the wash setup. Notably, and in agreement with the literature, GS-441524 is a highly selective inhibitor of SARS-CoV-2 Mac1 with slight activity as well on SARS-CoV and CHIKV Mac1 (Figure 2B). When comparing potency, the IC_50_ determined for SARS-CoV-2 Mac1 (105 μM) is approximately seven times higher than that described by Anmangandla et al. as determined in a tracer displacement assay (15.2 μM).^23^ In contrast to the latter, our assay relies on the enzymatic processing of MARylated PARP10cat. That means the deMARylated fraction of PARP10 will decrease over time whenever there is even the slightest remaining hydrolyzing activity of Mac1. As our assay reports on the %-share of remaining MARylated substrate, this results in a tendency for higher IC_50_ values compared to approaches based on displacement of a not hydrolysable tracer.

We then examined the tryptophan derivative 15c which is the most potent SARS-CoV-2 Mac1 inhibitor described by Sherrill et al.^39^ In this publication, 15c is tested for its binding to SARS-CoV-2 Mac1 using an AlphaScreen and an FRET displacement assay, but also for its ability to inhibit the enzymatic activity of Mac1 in the ADPr-Glo assay. While IC_50_ values of about 10 μM are reported for the two binding assays, the IC_50_ for the enzymatic ADPr-Glo assay is not mentioned, but data suggest a value above 100 μM. This is in agreement with the IC_50_ of approx. 200 μM determined in our wash setup (Figure 2B). Like GS-441524, 15c is highly selective for SARS-CoV-2 Mac1 with some additional weak activity on SARS-CoV and MERS-CoV Mac1 that is too low to determine proper IC_50_ values. On the other hand, 15c shows distinct quenching of the readout system in the one-pot setup, implying that 15c addresses PARP14 Mac 2/3 as an off-target.

**Figure 3:**
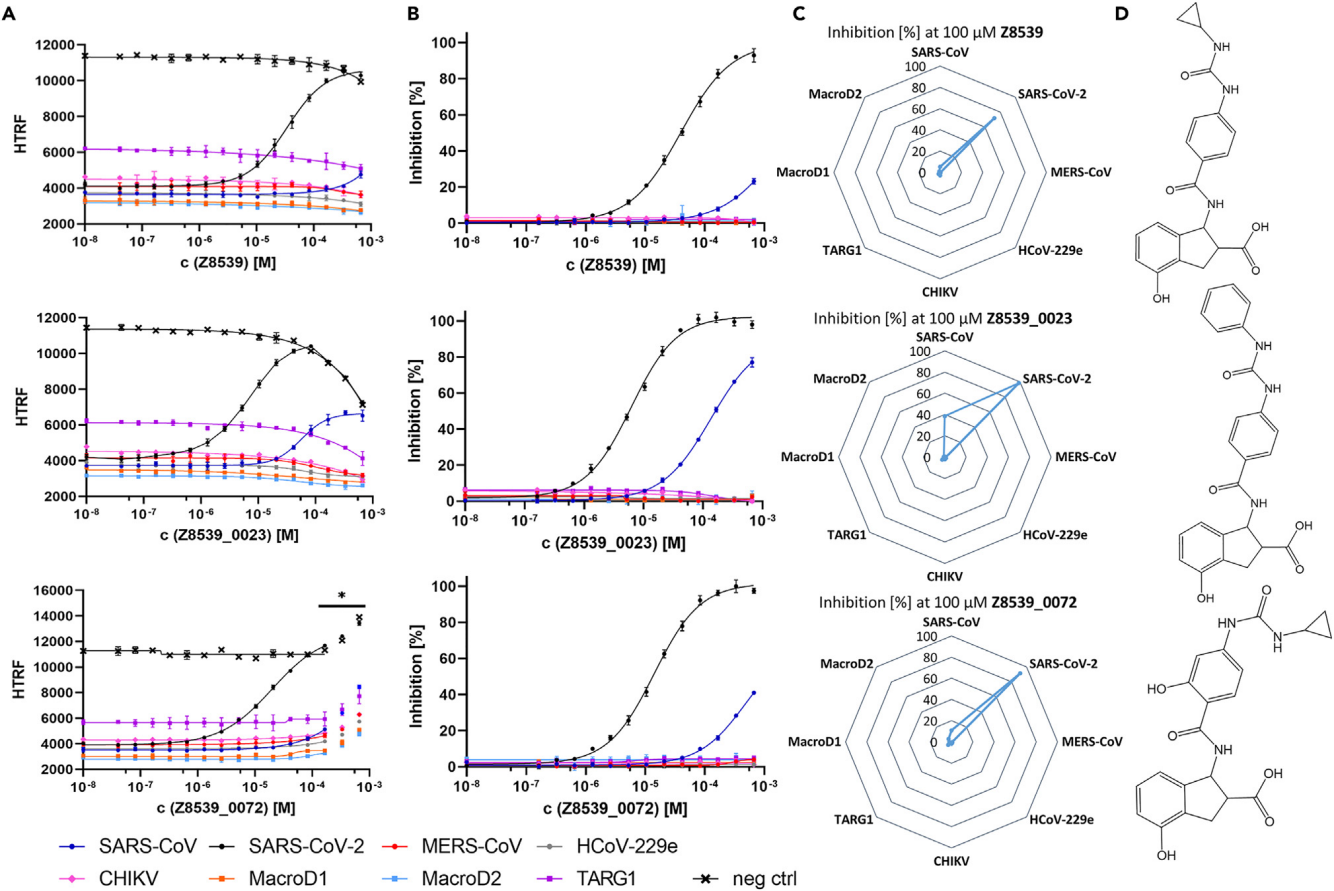
Examination of the inhibitory potential of Z8539, Z8539_0023, Z859_0072 on eight different Mac1 homologs (A) One-pot setup: Each compound was titrated up to 667 μM to 3 nM Tb-PARP10cat, and a defined Mac1 concentration; for SARS-CoV (*dark blue*), SARS-CoV-2 (*black*), MERS CoV (*red*), HCoV-229e (*gray*) 80nM, for CHIKV (*pink*) 300 nM, for TARG1 (*purple*) 2.5 μM, and for MacroD1 (*orange*), MacroD2 (*light blue*) 10 nM. The readout was performed with 200 nM sGFP-PARP14 Mac2/3 as described in STAR Methods. A negative control without Mac1 homolog addition was included in each experiment. A DMSO control is the lowest concentration in each dataset. Data represent the means ± SD of 3 technical replicates for each construct. R^2^ for each curve equals >99%. The inhibitory effect of Z8539_0023 on SARS-CoV2 was curve fitted with only concentrations below 100 μM being considered; higher concentrations are shown with a connecting line instead. Z8539_0072 led to substantial quenching of Tb-cryptate at concentrations above 167 μM, resulting in abnormally high HTRF values; these values were therefore excluded from the curve fitting and are marked with an asterisk. (B) Wash setup: Each compound was titrated up to 667 μM to 6 nM immobilized Tb-PARP10cat, and a defined Mac1 concentration (as described in (A)). The readout was performed with 200 nM sGFP-PARP14 Mac2/3 after washing as described in STAR Methods. Curves were fitted based on the HTRF signals and then normalized according to the respective upper and lower plateau. A DMSO control is the lowest concentration in each dataset. Data represent the means ± SD of 3 technical replicates for each construct. R^2^ for each curve equals >99%. (C) Radar plots were generated based on the data from the wash setup experiments to visualize the inhibition [%] at 100 μM of each compound tested for all eight macrodomains examined. (D) Structural formulas of the tested compounds; structures were created using ChemDraw.

**Figure 4:**
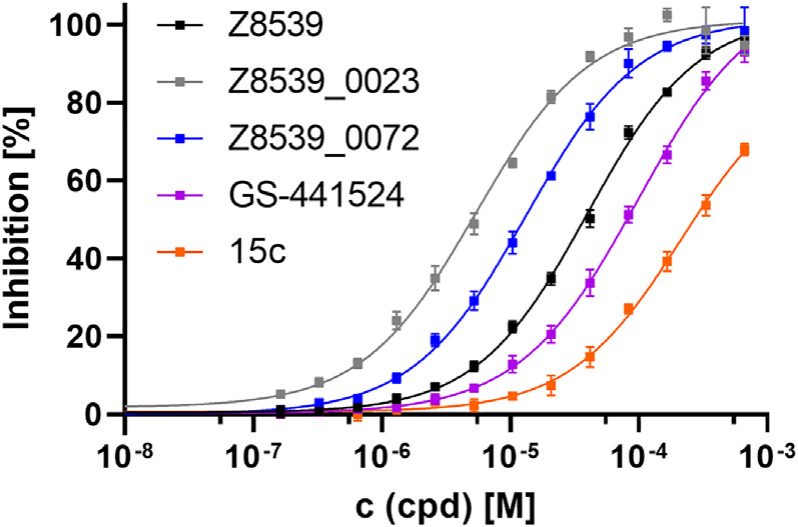
Direct comparison of the best performing compounds tested on SARS-CoV-2 Mac1 in the wash setup Each compound was titrated up to 667 μM to 6 nM immobilized Tb-PARP10cat, and 80 nM SARS-CoV-2 Mac1. The readout was performed with 200 nM sGFP-PARP14 Mac2/3 after washing as described in STAR Methods. Curves were fitted based on the HTRF signals and then normalized according to the respective upper and lower plateau. A DMSO control is the lowest concentration in each dataset. Data represent the means ± SD of 3 technical replicates for each construct. R2 for each curve equals >99%.

Furthermore, we became aware of a recent publication by Gahbauer et al.^26^ reporting the structure-based discovery and development of several different chemical scaffolds with sub-micromolar affinity for SARS-CoV-2 Mac1. From this publication we purchased the three most potent compounds Z8539, Z8539_0023 and Z8539_0072 (Enamine) and tested them in the deMARylation assay (Figure 3). In fact, all three compounds are highly active against SARS-CoV-2 Mac1 and have significantly lower IC_50_ values compared to GS-441524 and 15c (40 μM, 6 μM and 14 μM, respectively), in particular Z8539_0023 is about twenty times more potent than GS-441524 (Figure 4; Table 2). However, the IC_50_ values obtained are again approximately 10–30 times higher than those reported by Gahbauer et al., again demonstrating that IC_50_ values determined by an enzymatic assay are only partially comparable to IC_50_ values determined by a displacement assay; (Table 3). Nevertheless, these results demonstrate that it is possible to identify more potent inhibitors for SARS-CoV-2 Mac1 than free ADPr. Apart from this, like the previously described GS-441524 and 15c, all three Z8539 derivatives are highly selective for SARS-CoV-2 Mac1, while also showing some affinity for SARS-CoV Mac1. Especially the phenylurea derivative Z8539_0023 has a comparatively high activity against SARS-CoV Mac1 with an IC_50_ of 135 μM. Interestingly, the parent compound Z8539 is the only compound tested so far that does not show a strong quenching effect in the one-pot setup, suggesting that Z8539 has no affinity for PARP14 Mac2/3. On the contrary, the most potent inhibitor Z8539_0023 leads to pronounced readout quenching, while the reference FI of the FRET donor was unaffected. This suggests that the phenyl-phenyl urea derivative lost its advantageous selectivity against the reader macrodomains of PARP14.

**Table 2:** IC_50_ values for compounds tested in wash setup

IC_50_ [μM]	SARS-CoV	SARS-CoV-2	MERS-CoV	HCoV-229e	CHIKV	TARG1	MacroD1	MacroD2
ADPr	65	66	60	Unq.^a^	110	Unq.^a^	2	0.5
GS-441524	Unq.^a^	105	/	/	Unq.^a^	/	/	/
15c	Unq.^a^	203	Unq.^a^	/	/	/	/	/
Z8539	Unq.^a^	40	/	/	/	/	/	/
Z8539_0023	135	6	/	/	/	/	/	/
Z8539_0072	**Unq.**^a^	**14**	**/**	**/**	**/**	**/**	**/**	**/**

a Unquantifiable.

**Table 3:** Comparison of in literature reported IC_50_ values with tested IC_50_ values for SARS-CoV-2 Mac1

IC_50_ [μM]	This study	Ref.^23^	Ref.^39^	Ref.^39^	Ref.^39^	Ref.^26^
	**DeMAR-HTRF** **(wash protocol)**	**TAMRA-ADPr** **FP**	**AlphaScreen™**	**GAP FRET**	**ADPr-Glo**	**HTRF (peptide displacement)**
GS-441524	105	15	/	/	/	/
15c	203	/	6	11	>100	/
Z8539	40	6	/	/	/	1
Z8539_0023	6	/	/	/	/	0.5
Z8539_0072	**14**	**/**	**/**	**/**	**/**	**0.4**

Anmangandla et al.^23^

Sherrill et al.^39^

Gahbauer et al.^26^

**Figure 5:**
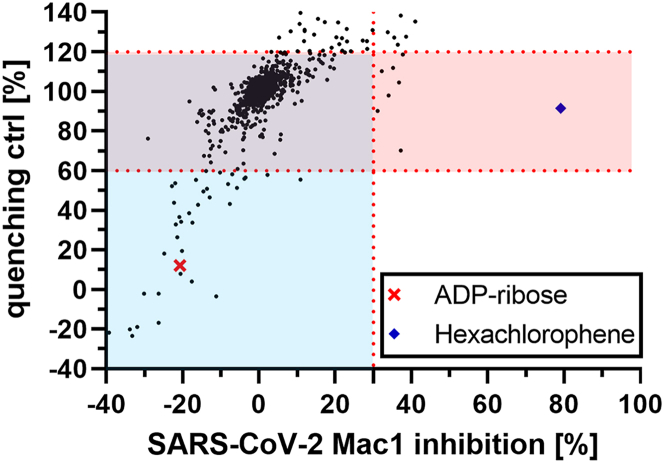
Evaluation of the SARS-CoV-2 Mac1 Prestwick library screen A total of 2528 compounds plus the respective control were screened. The screen was performed according to one-pot protocol as described in STAR Methods with a compound concentration of 100 μM for approved drugs and 250 μM for fragments. The scatterplot shows for each compound in the screen the Mac1 inhibition [%] (x axis) against the counter screen (no Mac1) readout quenching control [%] (y axis). 100% in the quenching control indicates 100% HTRF signal with 0% signal loss in the counter screen. Compounds with minimum SARS-CoV-2 Mac1 inhibition of 30% and 60–120% quenching ctrl were identified as hits. In addition, the donor FI quenching was not allowed to exceed 70–130%. Compounds in the *red* area are characterized as Mac1 hits, while compounds in the *light blue* area represent PARP14-binding readout quenchers. Compounds in the *violet* area are not active in either way. The average Z′ score was 0.91 ± 0.01.

We then adapted the one-pot protocol by introducing an Echo 550 acoustic dispenser (Beckman Coulter) for compound presentation so that the protocol could be used for high-throughput screening. Subsequently, we performed a pilot screen on SARS-CoV-2 Mac1 of the FDA-approved drugs library (Prestwick 3 Collection; 1280 compounds), a subset of the Prestwick drug-fragments library (480 compounds) as well as the DSi-P library (Diamond-SGC-iNEXT library of fragments poised for eased follow-up synthesis; 768 compounds) (Figure 5). Approved drugs were screened at 100 μM, while fragments (MW < 300 Da) were tested at 250 μM. In addition to the on-target screen, each compound was also subjected to a counter-screen without macrodomain to identify compounds that also target PARP14 Mac2/3. Hits (*red area*) were defined as compounds that inhibited Mac1 by more than 30%, that were within 60%–120% of readout quenching ctrl., and for which FRET donor FI was almost unaffected, within 70%–130% of controls. In terms of readout quenching, 100% represents a 100% HTRF signal with 0% signal loss in the counter screen without Mac1, and the upper threshold of 120% was chosen to be particularly restrictive in order to filter out aggregators that might show up as false positives. Using these hit criteria, we identified three fragments and five approved drugs resulting in a 0.32% hit rate. After performing dose-response experiments in the one-pot and the wash setup (Figures S5 and S6), we were able to confirm hexachlorophene as a SARS-CoV-2 Mac1 inhibitor with an IC_50_ of 114 μM but limited efficacy, probably due to its low solubility in aqueous conditions. Unfortunately, hexachlorophene, once a widely used disinfectant, has been banned from consumer products worldwide due to neurotoxicity and is therefore not of interest for further antiviral research.^40^ Apart from this, we have identified several compounds that primarily target PARP14 Mac2/3 with a potency similar to that of free ADPr (*blue area*). However, because we do not want to address this important off-target, we did not investigate these compounds further. Besides this, a Z′ score of 0.91 ± 0.01 was calculated based on all DMSO controls with and without Mac1, which were conducted in the screen, demonstrating that the one-pot deMARylation assay is highly suitable for high-throughput screening.

## Discussion

The outbreak of SARS-CoV-2, which is the third beta coronavirus to spread among humans in the last three decades, has exposed a shortage of antivirals that can target coronaviruses and other viruses with pandemic potential. In addition, COVID-19 remains a threat for vulnerable populations and causal treatment options for Long-/Post-COVID conditions pose a high unmet medical need. In this context, Mac1 of SARS-CoV-2 represents an attractive therapeutic target as it is critical for viral pathogenesis and functionally as well as structurally distinct from commonly addressed targets such as viral proteases and polymerases.

To help facilitate the drug discovery process for this promising target, we present here the development of an assay system that is based on HTRF, a technique widely used for HTS. The deMARylation assay exploits the well-characterized interaction between MARylated PARP10 catalytic domain and PARP14 Mac2/3.^27^^,^^28^^,^^29^ PARP10 is highly upregulated during CoV infection, and for CHIKV, PARP10-mediated MARylation has been shown to inhibit viral replication by preventing polyprotein processing.^4^^,^^11^ In turn, Grunewald et al.^4^ were able to show that PARP14 is required to induce increased IFN-β production during coronavirus infection and is also essential to inhibit the replication of a Mac1-deficient MHV coronavirus. However, although suggested by the functional interplay of PARP10 and PARP14, there are actually only limited data supporting a significant role of the direct interaction of MARylated PARP10 and PARP14 Mac2/3 in the antiviral response. Nevertheless, PARP10cat is one of the very few proteins for which the exact position of mono-ADP-ribosylated residues has been determined, of which E882 was found to be a major, but not the only, acceptor site for PARP10 auto-MARylation.^29^^,^^41^ Moreover, as the PARP10:PARP14 interaction is strictly dependent on MARylation, the PARP10cat:PARP14 Mac2/3 interaction can be deemed prototypical for MAR-dependent protein-protein interactions (PPIs) involving reader macrodomains.^27^ The fact that MARylated PARP10cat is a suitable substrate for viral macrodomains qualifies it as a particularly interesting assay concept. Most other biochemical assays described in the literature are based on ADP-ribose conjugated tool peptides that cannot be deMARylated and serve only as tracers in displacement assays. In contrast, the ADPr-Glo assay published by Dasovich et al.,^25^ uses as substrate a fusion protein composed of SUMO (small ubiquitin-like modifier) and a peptide from PARP10cat corresponding to the sequence surrounding E882. Finally, the assay system presented here uses the entire PARP10 catalytic domain as substrate and employs the MAR-dependent PARP10:PARP14 interaction for the detection strategy.

The deMARylation assay, together with the ADPr-Glo assay, is the only biochemical assay published to date that allows high-throughput screening for Mac1 inhibitors with a detection concept that reports on the inhibition of the enzymatic activity of Mac1. This is important because measuring inhibitor binding independently of enzyme activity may be attractive for its often economic and technical convenience, but on the other hand, inhibitor binding does not necessarily correlate with inhibition of catalytic activity.^42^ Furthermore, the risk of missing compounds targeting an allosteric site is higher with a displacement assay than with an enzymatic assay.^43^

The ADPr-Glo assay employs several enzymatic steps. At first, free ADP-ribose produced by the deMARylation activity of Mac1 is converted to AMP by the phosphodiesterase NudF, and in a second step, AMP is translated into luminescence using the commercially available AMP-Glo kit, which itself relies on several enzymes. In comparison, the DeMAR assay provides a more direct readout of the enzymatic activity of Mac1, as no additional enzymes are required. This is preferable because any additional enzyme involved in the setup will potentially lead to more false positive hits in a screen and therefore require additional control experiments. In this context, it should be emphasized that the PARP14 Mac2/3 counter screen without Mac1, which is performed as compound interference control for the one-pot setup, is a valuable off-target counter screen in its own right. MARylation is thought to either moderate PPIs or to modulate activity of the MARylated protein. In the case of MAR-driven PPIs, mono-ADP-ribose is the main binding epitope recognized by reader macrodomains in which ADPr occupies a specific binding pocket.^27^ As a result, free ADPr is able to block MAR-dependent PPIs, as reported by Ekblad et al.^28^ and as observed in our model system involving PARP14 Mac2/3 (Figure 2). Since ADPr is also the dominant substrate moiety recognized by the hydrolyzing viral macrodomains, inhibitors of the latter run the risk of also blocking the reader macrodomains of host factors.

Another advantage of the DeMAR assay is that the readout is completely independent of the macrodomain itself, so one could easily test the deMARylation activity of e.g., the entire nsp3. This modular aspect of the assay is also of interest in terms of pandemic preparedness, as it allows rapid adaptation to any new virus of concern that possesses a MAR hydrolyzing macrodomain, which accepts MARylated PARP10cat as substrate. This is also advantageous compared to a tracer-based displacement approach because a tracer will bind to different macrodomains with different affinities. Due to effects such as diffusion enhanced FRET, aggregation, and fluorophore auto-quenching, the tracer concentration cannot necessarily be adjusted accordingly, which may prevent an appropriate assay window. This phenomenon can be observed in Anmangandla et al.^23^ where distinctly smaller mP shift values were reported for the macrodomains of CHIKV and VEEV compared to MacroD1 and MacroD2, caused by lower binding affinity to the FP tracer, TAMRA-ADPr.

In conclusion, the DeMAR assay system represents a functional assay that complements existing screens and will be a valuable tool for the identification and characterization of viral macrodomain inhibitors. Targeting viral macrodomains is an approach to antiviral drug development that is highly attractive because there is strong evidence that such inhibitors may not only reduce viral replication but also restore innate antiviral host defense mechanisms.^13^^,^^14^ The modular design of the deMARylation assay also makes the assay easily adaptable for screening different macrodomains, taking into account that the macrodomain fold is highly conserved in both coronaviruses and alphaviruses. In this study, we demonstrated that the established assay is suitable for high-throughput screening in 384-well format. Although our pilot screen on SARS-CoV-2 did not identify a suitable novel lead compound, we were able to demonstrate that none of the 1280 FDA-approved drugs included in the Prestwick panel is a candidate for repurposing. However, using the deMARylation assay, we evaluated the most promising Mac1 inhibitors in recent literature. Especially for the compounds published by Gahbauer et al.^26^ it was encouraging to see that all three compounds tested show surprisingly high selectivity for SARS-CoV-2 Mac1. We consider it is essential that Mac1 inhibitors, in addition to substantial structural deviations from ADPr, have sufficient off-target selectivity against MAR-interacting host factors such as hydrolyzing as well as reader macrodomains. Although considerable efforts have been made, the identification and development of nanomolar inhibitors, which are likely to be required for *in vivo* studies, remains a challenge and further research in this area is essential, which we hope will be facilitated by the tools presented.

### Limitations of the study

The assay system presented here is specifically designed for macrodomains with mono-ADP-ribosyl hydrolase activity. However, macrodomains that do not accept MARylated PARP10cat as a substrate cannot be assayed.

The assay takes advantage of the enzymatic activity of the macrodomain under study, and achieved good Z′ with SARS-CoV-2 Mac1, qualifying it for HTS. However, since the substrate is not in excess of the macrodomain, the assay does not allow the study of enzyme kinetics. If such is intended, we would suggest to try the ADPr-Glo assay.^25^

Off-target activity against the host proteins MacroD1, MacroD2 and, to a limited extent, also TARG1 can be tested. On the other hand, PARG (poly(ADP-ribose) glycohydrolase), an essential enzyme that removes poly-ADP-ribose from substrates, cannot be tested in the DeMAR assay. PARG as well as reader macrodomains of host factors, must be considered as potential off-targets in order to avoid unwanted, potentially toxic effects. As thoroughly discussed above, free ADPr is also bound by the PARP14 reader macrodomains that are employed in the readout strategy of the presented assay system. In the one pot protocol ADPr and other compounds that address PARP14 Mac2/3 will cause quenching of the HTRF signal. Consequently, such scaffolds can only be tested for Mac1 inhibition in the wash protocol.

## STAR★Methods

### Key resources table

**Table:** 

REAGENT or RESOURCE	SOURCE	IDENTIFIER
**Bacterial and virus strains**		

NEB® 5-alpha competent *E. coli*, DH5α™ derivative	NEB	Cat# C2987
T7 express competent *E. coli*, BL21 DE3 derivative	NEB	Cat# C2566

**Chemicals, peptides, and recombinant proteins**		

Q5® high-fidelity DNA polymerase	NEB	Cat# M0491L
DpnI restriction endonuclease (template DNA removal)	NEB	Cat# R0176L
BamHI-HF restriction endonuclease	NEB	Cat# R3136L
XhoI restriction endonuclease	NEB	Cat# R0146L
NEBuilder® HiFi DNA assembly master mix	NEB	Cat# E2621L
T4 DNA ligase	NEB	Cat# M0202S
SNAP-Lumi4-Tb labeling reagent; for labeling with terbium cryptate via SNAP-tag	cisbio	Cat# SSNPTBD
EDTA-free cOmplete™ protease inhibitor cocktail tablets	Roche	Merck Cat# 11873580001
Prestwick chemical Library®: FDA-approved & EMA-approved drugs for HTS screening	Prestwick	Prestwick-chemical-library
Prestwick chemical Library®: FRACS	Prestwick	Prestwick-chemical-library
DSI library	Diamond Oxford	N/A
β-Nicotinamide adenine dinucleotide hydrate	Sigma Aldrich	Cat# N6522
Adenosine 5′-diphosphoribose sodium salt	Sigma Aldrich	Cat# A0752

**Oligonucleotides**		

58_pBH4-6H-TEV_BamHI.rev: 5′- CCCCGGATCCCTGGA AGTACAGGTTTTCG-3′	This paper	N/A
527_SNAPt2_BamHI.rev CCCCGGATCCCAGACCCGGTTTACCCAGACGATGAC CTTCATGGGC	This paper	N/A
600_pBH4-3’end_HindIII_XhoI.for TTAGAAGCTTCTCGAGATCCGGCTGCTAACAAAGC	This paper	N/A
626_insert Avitag.for: 5′- GACTACCGAAAACCTGTACTT CCAGGGTCTGAACGACATCTTCGAGGCTCAGAAAATC GAATGGCACGAAGG-3′	This paper	N/A
919_Avitag_kpnI.rev: 5′- CCCCGGTACCTTCGTGCCATT CGATTTTCTGAGC-3′	This paper	N/A
929_ARTD8_Mac2_BamHI.for: 5′-CACATGGCATGGATGAGC TCTACAAAGGATCCGCAGCAGCCGGTCCTGGTAAAACC-3′	This paper	N/A
930_ARTD10_cat_BamHI.for: 5′-GGGTAAACCGGGTC TGGGATCCAGCGGTCCGACACTGGCAG-3′	This paper	N/A
931_ARTD10_cat_XhoI.rev: 5′- GCTTTGTTAGCAGCCGG ATCTCGAGTCTAATCATTTAGGTATCCGGACTACGAC CAGGCAG-3′	This paper	N/A
939_gib_ARTD8_Mac3.rev: 5′- GCTTTGTTAGCAGCCGG ATCTCGAGTCATCTAATCATTTAGCTGCTCAGCTGGG TGCCTTC-3′	This paper	N/A
1010_SNAP_KpnI.for: 5′- CCCCGGTACCGACAAAGATTG CGAAATGAAACGTACCACCCTGG-3′	This paper	N/A

**Recombinant DNA**		

pET29BH4-SNAP	Hartmann et al.^44^	N/A
pET29BH4-sGFP	Hartmann et al.^44^	N/A
pET29BH4	Hartmann et al.^44^	N/A
SNAP-PARP10cat in pET29BH4	This paper	CM009
Avi-SNAP-PARP10cat in pET29BH4	This paper	CM012
sGFP-PARP14 Mac2/3 in pET29BH4	This paper	CM008
[SARS-CoV2; "Uniprot: P0DTD1" res. 1022-1197] in pET29BH4	This paper	CM003
[SARS1; "Uniprot: P0C6X7" res. 998-1177] in pET29BH4	This paper	CM016
[CHIKV; "Uniprot: Q8JUX6" res. 1329-1496, mutant C1333S] in pET29BH4	This paper	CM013
[*h.s.* MacroD1, "Uniprot: Q9BQ69" res. 90-325] in pET29BH4	This paper	CM018
[*h.s.* MacroD2; "Uniprot: A1Z1Q3" res. 4-243] in pET29BH4	This paper	CM019
[*h.s.* TARG1; "Uniprot: Q9Y530" res. S3-152] in pET29BH4	This paper	CM020
[MERS; "Uniprot: K9N638" res. 1107-1275] in pET29BH4	This paper	CM017
[HCoV-229E; "Uniprot: P0C6X1" res. E1270-1437] in pET29BH4	This paper	CM014

**Software and algorithms**		

GraphPad PRISM, version 9; dose-response inhibition -log(inhibitor) vs. response with variable slope (four parameter fit)	GraphPad Software Inc.	Graphpad PRISM version 9
ChemDraw	Revvity	ChemDraw Pro
Microsoft® Excel® LTSC MSO (16.0.14332.20438)	Microsoft	Office 2021

**Other**		

Ni Sepharose® 6 fast flow	Cytiva	Sigma-Aldrich Cat# GE17-5318-02
HiLoad® 16/600 Superdex® 75 pg	Cytiva	Sigma-Aldrich Cat# GE28-9893-33
Superdex® 75 10/300 GL	Cytiva	Sigma-Aldrich Cat# GE17-5174-01
Pierce™ Monomeric Avidin UltraLink™ Resin	Thermo Scientific™	Cat# 53146
Multidrop™ Combi Reagent Dispenser	Thermo Scientific™	Cat# 5840300
HydroSpeed™ plate washer equipped with 96/384 well indexing wash head	Tecan	N/A
White PS microplate, 384 well, F-bottom, small volume, high base, med. binding	Greiner Bio-One	Cat# 784075
Pierce™ Streptavidin Coated High-Capacity Plates	Thermo Scientific™	Cat# 15505
Tecan SPARK equipped with enhanced fluorescence module	Tecan	N/A
Branson 250 Digital Sonifier	Branson	N/A

### Resource availability

#### Lead contact

Further information and requests for resources and reagents should be directed to and will be fulfilled by the lead contact, Jan Heering (Jan.Heering@itmp.fraunhofer.de).

#### Materials availability

All unique/stable reagents generated in this study are available from the lead contact with a completed Materials Transfer Agreement.

#### Data and code availability

All experimental data required to reanalyze the data reported in this paper are available from the lead contact upon request. This paper does not report original code. Any additional information required to reanalyze the data reported in this paper is available from the lead contact upon request.

### Method details

#### Cloning

Plasmids for expression of PARP10cat with N-terminal SNAP-tag were cloned based on pET29BH4-SNAP^44^ that encodes an open reading frame (ORF) starting with Met-Gly-[His10]-Asp-Tyr-Asp-Ile-Pro-Thr-Thr followed by a cleavage site for Tobacco Etch Virus protease (TEV), a restriction site for KpnI (in frame translating to Gly-Thr with Gly being part of the TEV site), SNAP (residues 2-181), in frame BamHI site (Gly-Ser), and a XhoI site. The plasmid vector was amplified by PCR using Q5 DNA polymerase (NEB) with oligos #527 and #600, then digested with DpnI, BamHI and XhoI, and purified using QIAquick PCR purification kit. The coding DNA sequence (CDS) for *h.s.* PARP10 catalytic domain (PARP10cat; "Uniprot: Q53GL7") was codon optimized for *E.coli* and ordered as a synthetic dsDNA string from GeneArt (Regensburg, Germany). CDS of PARP10cat (residues 840-1025) was amplified by PCR using oligos #930 and #931 that introduced overhangs matching to SNAP CDS (upstream) and vector backbone (downstream) which enabled insertion of the PARP10cat insert into pET29BH4-SNAP between BamHI and XhoI via Gibson assembly using NEBuilder HiFi DNA Assembly Master Mix (NEB) resulting in the plasmid SNAP-PARP10cat in pET29BH4. The encoded ORF reads as follows; with amino acid sequence in single letter code, tags and proteins in brackets, ∗ indicates TEV cleavage: MG-[His10]-DYDIPTT ENLYFQ∗GT-[SNAP res. 2-180]-GS-[PARP10cat res. 840-1025]

The wash protocol requires coupling of PARP10cat via biotin:streptavidin. For this purpose, an additional Avi-tag was introduced between TEV site and SNAP-tag. In the first five cycles of a PCR reaction primer #919 was elongated against oligo #626 (5:1 M equivalent). Then oligo #1010 was added together with before described plasmid. Elongated #919 together with #1010 then produced a linear amplificate with KpnI sites on both ends. After digest with DpnI and KpnI, and purification the plasmid was re-ligated using T4 DNA ligase resulting in the plasmid Avi-SNAP-PARP10cat in pET29BH4. The ORF reads as follows:

MG-[His10]-DYDIPTTENLYFQ∗GLNDIFEAQKIEWHEGT-[SNAP res. 2-180]-GS-[PARP10cat res. 840-1025]

PARP14 macrodomains 2 and 3 were cloned into the recently described pET29b derivative pET29BH4-sGFP that encompasses the CDS for superfolder GFP (sGFP; residues 2-238, addgene #6471).^44^^,^^45^ The plasmid vector was digested with BamHI and XhoI, and gel purified. The coding DNA sequence (CDS) for *h.s.* PARP14 macrodomain 2 and 3 (PARP14 Mac2/3; "Uniprot: Q460N5-6", residues 994-1393) was codon optimized for *E.coli* and ordered as a dsDNA string (GeneArt). The CDS was amplified by PCR using oligos #929 and #939 that introduced overhangs matching to sGFP CDS (upstream) and vector backbone (downstream) enabling Gibson assembly of the PARP14 Mac2/3 insert into pET29BH4-sGFP between BamHI and XhoI resulting in the plasmid sGFP-PARP14 Mac2/3 in pET29BH4. The encoded ORF reads as follows:

MG-[His10]-DYDIPTTENLYFQ∗GSA-[sGFP res. 2-238]-GS-[PARP14 Mac2/3 res. 994-1393]

The viral and human macrodomains were cloned into pET29BH4 which was PCR amplified using oligos #58 and #600, thereafter digested with DpnI, BamHI and XhoI, and purified. The CDS for the macrodomains under investigation were codon optimized for *E.coli* and ordered as dsDNA strings (GeneArt) with overhangs matching the flanking sequences of the vector in order to enable Gibson assembly. Thereby the 5′ sticky end of the cleaved BamHI site of the vector is removed which made the second codon after the TEV cleavage site free for adaptation to the macrodomain protein sequences. In the encoded ORFs the protein sequence MG-[His10]-DYDIPTTENLYFQ∗G precedes [SARS-CoV-2; "Uniprot: P0DTD1" res. 1022-1197], [SARS-CoV; "Uniprot: P0C6X7" res. 998-1177], [CHIKV; "Uniprot: Q8JUX6" res. 1329-1496, mutant C1333S], [*h.s.* MacroD1, "Uniprot: Q9BQ69" res. 90-325], [*h.s.* MacroD2; "Uniprot: A1Z1Q3" res. 4-243], [*h.s.* TARG1; "Uniprot: Q9Y530" res. S3-152]. And MG-[His10]-DYDIPTTENLYFQ∗GT precedes [MERS; "Uniprot: K9N638" res. 1107-1275], and [HCoV-229E; "Uniprot: P0C6X1" res. E1270-1437].

DH5α competent *E.coli* (NEB) were transformed, plasmids prepared using NucleoSpin Plasmid (Macherey-Nagel), and plasmids were verified by overlapping sanger sequencing of the entire ORF, respectively.

#### Heterologous protein expression in *E. coli*

For the expression of all proteins utilized, expression constructs (pET-based plasmids) were transformed into competent *E. coli* T7-express cells (New England Biolabs, NEB; #C2566). Except for the expression of the macrodomain homologs, the cells were co-transformed with pGro7 for co-expression of GroEL/ES (chaperone plasmid set; TaKaRa Bio, Inc.) in order to support protein folding. After transformation the cells were selected overnight at 37°C on Luria Broth (LB) agar containing 35 μg/mL kanamycin for pET constructs and, if necessary, 34 μg/mL chloramphenicol for pGro7. The grown bacterial colonies were then transferred into 250 mL LB-medium containing the respective antibiotics. After 1–2 h in an incubation shaker at 37°C and 180 rpm, expression cultures of 1 L LB-medium were started with approximately 40 mL preculture and then grown at 37°C and 180 rpm. If co-transformation with pGro7 was conducted, GroEL/ES chaperone expression was induced with 1 g/L L(+)-arabinose once the optical density at 600 nm (OD_600_) had reached 0.6 to 0.7. Concurrently, temperature was lowered to 20°C and shaking was reduced to 120 rpm. After about 30 min later, when the OD_600_ approximately equaled 1.0, the target protein expression was induced by the addition of 0.5 mM IPTG, and cultures were supplemented with ∼200 μL Antifoam Y-30 (Sigma-Aldrich # A5758) per liter to ensure good aeration. Expression cultures were incubated overnight and harvested at 4°C at 6000 rpm. The corresponding cell pellets were either stored at −80°C or processed immediately.

For cell lysis, pellets of 2 L bacterial culture were resuspended on wet ice in 50 mL buffer A (400 mM NaCl, 20 mM NaPi pH 7.8, 10% (w/v) glycerol, 20 mM β-mercaptoethanol) supplemented with 20 mM imidazole, 2 mM MgSO_4_, 750 Kunitz DNAse I (Sigma-Aldrich #D5025), 250 Kunitz RNAse A (Sigma-Aldrich #R5503) and 1x EDTA-free cOmplete protease inhibitor cocktail (Roche AG). Subsequently, cells were disrupted by addition of lysozyme (Sigma-Aldrich #L6876) and by sonication for 1 min at intervals of 1 s pulsing with an amplitude of 70% and 3 s pause. In case GroEL/ES was co-expressed the lysed suspension was additionally incubated with 1 mM ATP for 1 h on wet ice in order to ensure the energy dependent release of folded target protein from the chaperone. After lysis, cell debris was removed by centrifugation at 16,500 x g for 20 min at 4°C.

#### Purification

As a first purification step for all His-tagged fusion proteins an immobilized metal affinity chromatography (IMAC) was performed on an ÄKTA purifier FPLC system (GE Healthcare) equipped with a monochromator (254, 280, 495 nm) using columns packed with Ni Sepharose 6 Fast Flow resin (Cytiva). Therefore, the loaded His-tagged proteins were initially washed for 15 column volumes with buffer A supplemented with 25 mM imidazole and then eluted with 250 mM imidazole. In the following, a TEV digest was conducted overnight with His-tagged TEV protease (1:50 M ratio). In parallel, the imidazole content was reduced to 10 mM by dialysis against buffer A, allowing the target protein to be separated from the cleaved His-tag and His-tagged TEV protease by reverse IMAC. Subsequently, the resulting flow through was concentrated in an Amicon stirred cell equipped with a 10,000 MWCO membrane (Merck) under nitrogen gas with 2 bar of overpressure. As a final purification step, a size exclusion chromatography (SEC) was performed. For this purpose, concentrates were applied to a 16/600 Superdex75 column equilibrated and run in high glycerol HTRF buffer (25 mM Hepes pH 7.5, 150 mM kF, 10% (w/v) glycerol, 5 mM DTT). The elution fraction was collected and then aliquots were prepared, which were frozen in liquid nitrogen and then stored at −80°C. After SEC, yield of proteins that did not need any further modification was in the range of approx. 5–20 mg/L of expression culture. To generate biotin-labeled Avi-SNAP-PARP10cat, the protein was site-specifically biotin-labeled via the introduced Avi-tag, and an additional affinity chromatography was performed using a column packed with 5 mL of monomeric avidin UltraLink resin (Pierce , ThermoScientific; #53146). Therefore, at first, the IMAC elution fraction was supplemented with His-tagged *E. coli* biotin ligase BirA in addition to the His-tagged TEV protease to ensure site-specific biotinylation of the Lys residue in the Avi-tag. Biotinylation and TEV digest were conducted in a dialysis setting overnight at 4°C against buffer A supplemented with 0.5 mM biotin, 0.5 mM ATP and 5 mM MgCl_2_. The dialysis against buffer A was then repeated several times to ensure that the biotin concentration was reduced to approximately ≤1 μM to maximize the capture efficiency of the following affinity chromatography step with monomeric avidin resin. After subjecting the protein solution to the packed column, unlabeled protein, TEV protease, and BirA biotin ligase were removed by washing with ten column volumes of buffer A before elution of the biotin-labeled protein in buffer A supplemented with 2 mM biotin. In this protocol the capacity of the monomeric avidin column is the limiting factor for yield and the flow through still contains a substantial amount. Henceforth, the column was regenerated according to the manufacturer’s instructions with 0.1 M glycine pH 2.8, and re-equilibrated in buffer A. Then the flow through was passed again through the column, and the purification repeated in total 3 times. Afterwards, the eluted protein was concentrated as described above, and then as a final purification step, a SEC was performed on a 10/300 Superdex75 column equilibrated and run in high-glycerol HTRF buffer.

#### MARylation of PARP10cat

For MARylation of PARP10cat the auto-MARylation capability of PARP10 catalytic domain (PARP10cat) was utilized. PARP10cat can only catalyze MARylation, but no PARylation, because its catalytic domain lacks the catalytic glutamate necessary to support the transition state during the enzymatic reaction and to elongate the ADP-ribose chain.^29^ Henceforth, a PARP10cat preparation can be used for specific auto-MARylation. Therefore, the flow through of the reverse IMAC purification step was supplemented with 2 mM DTT and a high molar excess of 1 mM β-NAD^+^ according to the protocol described by Alhammad et al.^15^ After an overnight incubation at 4°C, a SEC was carried out as described under ‘purification’. For the Avi-tagged version of PARP10cat the elution fraction of the monomeric avidin affinity chromatography was used for auto-MARylation instead of reverse IMAC flow through. The following SEC was then again conducted as described above. Protein yield was 3.5 mg from 4 L of culture; SEC fractions of 2.5 mL with 30 μM, which would theoretically be sufficient for 500,000 data points.

As a quality control, all protein preparations utilized in this study were analyzed on SDS-PAGE (Figure S7).

#### Labeling of PARP10 with terbium cryptate

In order to enable covalent coupling to a FRET donor fluorophore, the catalytic domain of PARP10 (PARP10cat) was expressed as a fusion protein with a SNAP-tag (NEB). The latter is a mutant of the DNA repair protein O6- alkylguanine-DNA alkyltransferase. SNAP reacts specifically and rapidly with benzylguanine (BG) derivatives, but introduced mutations prevent completion of the enzymatic reaction which in turn allows irreversible covalent labeling of fusion proteins that incorporate the 20 kDa SNAP-tag. SNAP-Lumi4-Tb labeling reagent (cisbio) was used as substrate for labeling with terbium cryptate. A labeling rate of approx. 80% was aimed for in order to avoid free unconsumed SNAP-Lumi4-Tb remaining without the need of additional purification steps. According to this, a labeling reaction contained 600 nM SNAP-PARP10cat fusion protein in high glycerol HTRF buffer supplemented with 5% DMSO and 500 nM SNAP-Lumi4-Tb labeling reagent. After overnight incubation at 4°C, aliquots were prepared, which were flash frozen in liquid nitrogen and then stored at −80°C.

#### deMARylation assay in one-pot protocol

An HTRF assay was set up to investigate the potential inhibitory effects of low molecular weight compounds on viral macrodomains. In the build setup, the FRET donor (terbium cryptate) is coupled to MARylated PARP10cat. The MARylation on PARP10cat is able to recruit reader macrodomains such as those of PARP14. This assay utilizes the fusion protein sGFP-PARP14 Mac2/3, with N-terminal sGFP (superfolder green fluorescent protein) serving as FRET acceptor and the macrodomains 2 and 3 of PARP14 for specific binding of MAR. Binding of PARP14 Mac2/3 to MARylated PARP10cat results in the formation of a FRET productive complex with sGFP and terbium cryptate in close proximity. The deMARylation activity of a hydrolyzing macrodomain leads to a reduction in FRET, because PARP14 Mac2/3 does not recognize PARP10cat devoid of MARylated sites. Inhibiting the viral macrodomain using a potential inhibitor should protect PARP10cat from deMARylation, thereby preserving a high HTRF signal. In this setup, the concentrations of PARP10cat, PARP14 Mac2/3 as well as the viral macrodomain are kept constant while the potential inhibitor is titrated. The concentration of viral macrodomain varies depending on the macrodomain under investigation in order to account for its time-dependent deMARylation capacity. In case of SARS-CoV, SARS-CoV-2, MERS-CoV and HCoV-229e, the concentration was set at 80 nM, while the human macrodomains MacroD1 and MacroD2 were applied at 10 nM; the concentration used for CHIKV is 300 nM and for TARG1 2.5 μM. Experiments were performed in 384-well white non-treated polystyrene shallow-well small volume microplates (Greiner Bio-One #784075) and the reaction components were applied in three equal volumes of 5 μL each (1. PARP10cat, 2. test compound (incl. 4% DMSO), 3. macrodomain). Assay solutions were prepared in low glycerol HTRF assay buffer (25 mM Hepes pH 7.5, 150 mM kF, 5% (w/v) glycerol, 5 mM DTT) supplemented with 0.01% Triton X-100. At first, PARP10cat was pre-incubated for 15 min at room temperature (RT) with test compound or DMSO alone as negative control. Then the macrodomain was added, and the completed final reaction setup then contained 3 nM PARP10cat, macrodomain as listed above, and 1.33% DMSO with the concentration of the tested compound as indicated in the graphs, respectively. After incubation for 1 h at RT, the MAR-detection agent sGFP-PARP14 Mac2/3 was added to the reaction mixture, resulting in a final volume of 20 μL. The molar excess of 200 nM sGFP-PARP14 Mac2/3 versus 2.5 nM MARylated PARP10cat also blocked any further deMARylation by occupying all MARylated sites thereby shielding them from the hydrolyzing macrodomain. (No observable change in HTRF within 10 h.) After equilibration for 1 h at room temperature, measurements were performed on a Tecan Spark equipped with the enhanced fluorescence module. To address potential readout quenching by the tested compounds, a counter screen was always carried along under the same conditions, but without the hydrolyzing macrodomain.

For the compound library screen, compounds were dispensed using an Echo 550 acoustic dispenser (Beckman Coulter) at a compound concentration of 100 μM for approved drugs and 250 μM for fragments. Afterwards, PARP10cat and, 15 min later, the viral macrodomain were added manually in equal parts to match the concentrations of the reaction mix to the regular setup. As in the regular setup, after 1 h incubation at RT, detection agent sGFP-PARP14 Mac2/3 (200 nM) was added to a final volume of 20 μL and after another 1 h incubation for equilibration measurements were performed.

#### deMARylation assay as wash protocol

Since readout quenching by PARP14 Mac2/3 blockade was observed for some of the compounds tested, the one-pot protocol was adapted to include a wash step prior to addition of sGFP-PARP14 Mac2/3. This required stable coupling of PARP10cat to the assay plates. For this purpose, streptavidin-coated white 384-well microtiter plates (Thermo Scientific; #15505) were chosen, and a biotinylated version of SNAP-PARP10cat (biotin-labeled Avi-SNAP-PARP10cat; in short: biotin-PARP10cat) was expressed, biotin-labeled with BirA, purified on monomeric avidin, auto-MARylated, purified over SEC, and working stocks were labeled with terbium cryptate via the SNAP-tag. Prior to coupling, streptavidin-coated microtiter plates were initially washed four times with low glycerol HTRF assay buffer to remove commercially pre-applied blocking buffer using a HydroSpeed plate washer (Tecan), leaving 10 μL of buffer in each well. Subsequently, 5 μL of a 30 nM biotin-PARP10cat solution was added and incubated for 2 h at RT for immobilization by binding to streptavidin, before unbound protein was removed by washing four times, leaving 10 μL of buffer in each well. Then, according to the one-pot protocol, the viral macrodomain and the dilution series of inhibitor were combined to a final reaction volume of 25 μL containing 1.33% DMSO and the tested compound as well as the investigated macrodomain at the concentration indicated in the graphs, respectively. After incubation for 2 h at room temperature, the reaction was stopped by washing four times with HTRF assay buffer to remove the viral macrodomain, the tested compound, and also cleaved ADP-ribose. Following this, 10 μL of a 400 nM the MAR detection reagent sGFP-PARP14 Mac2/3 were added, resulting in a final volume of 20 μL with 200 nM sGFP-PARP14 Mac2/3 and the remaining coupled PARP10cat. Finally, the HTRF readout was performed on a Tecan Spark after equilibration for 1 h at room temperature.

#### Measurement of HTRF

After excitation at 340 nm, fluorescence intensities (FIs) at 520 nm (acceptor) and 620 nm (donor reference) were recorded on a Tecan Spark (Tecan). Measurements were conducted with 50 flashes, an integration time of 400 μs, and a lag time of 100 μs. For maximum sensitivity, the gain was usually set to optimal, while the experiments to be compared were measured at the same gain.

#### Calculation of HTRF

FI_520nm_ was divided by FI_620nm_ and multiplied by 10,000 to obtain dimensionless HTRF values as shown below.

$$HTRF=\frac{acceptor\;FI\cdot 10,000}{donor\;FI}$$

#### Curve fitting

To generate IC_50_ curves, data analysis was performed using GraphPad Prism v.9 (GraphPad Software, Inc.). Lower and upper plateaus, EC_50_ or IC_50_ values, as well as the hill slope were determined by nonlinear regression utilizing a variable slope equation (four parameters).

### Quantification and statistical analysis

For quantification of inhibition [%], the HTRF data presented in Figures 2, 3, and 4 were normalized using GraphPad Prism v.9 (GraphPad Software, Inc.). Control experiments with no Mac1 (100% inhibition, upper plateau) or Mac1 with only DMSO but no inhibitor being present (0% inhibition, lower plateau) were used as references.
