# Supplementary material for: HTRF-based assay for detection of mono-ADP-ribosyl hydrolyzing macrodomains and inhibitor screening
Source: iScience. 2024 Jun 20;27(7):110333. doi: 10.1016/j.isci.2024.110333 (PMC11269945; doi:10.1016/j.isci.2024.110333)
Supplement: Document S1. Figures S1–S7 [file mmc1.pdf]

iScience, Volume *27*

Supplemental information

HTRF-based assay for detection of mono-ADP-ribosyl hydrolyzing macrodomains and inhibitor screening

Niklas Ildefeld, Dieter Steinhilber, Ewgenij Proschak, and Jan Heering

SUPPLEMENTAL INFORMATION

Figure S1: Evaluation of the readout system with different Tb-PARP10cat concentrations.... 2

Figure S2: Evaluation of the influence of different glycerol and DMSO concentrations on the activity of SARS-CoV-2 Mac1. 3

Figure S3: Comparison of the activity of SARS-CoV-2 Mac1 with two inactive mutants......... 4

Figure S4: Evaluation of the influence of different incubation times on the deMARylation of SARS-CoV-2 Mac1 in the one-pot and in the wash setup.. 5

Figure S5: DeMAR assay (one-pot setup) dose-response experiments of the eight hits from the Prestwick SARS-CoV-2 Mac1 pilot screen.. 6

Figure S6: DeMAR assay (wash setup) dose-response experiments of the eight hits from the Prestwick SARS-CoV-2 Mac1 pilot screen. 7

Figure S7: SDS-PAGE analysis of all of the protein preparations that were used for the DeMAR assay. 8

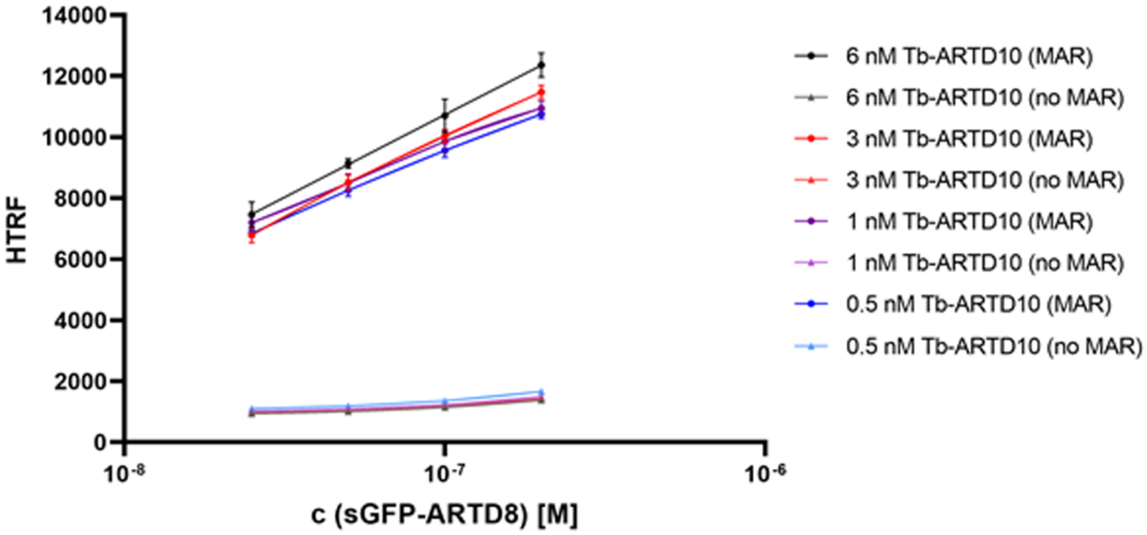

Figure S1: Evaluation of the readout system with different Tb-PARP10cat concentrations, related to Figure 1.
Reader sGFP-PARP14 Mac2/3 was titrated from 25 up to 200 nM against either 0.5, 1, 3, or 6 nM of MARylated (dot) or unmodified (triangle) Tb-PARP10cat. Data represent the means ± SD of 8 technical replicates for each Tb-ARTD10cat concentration.

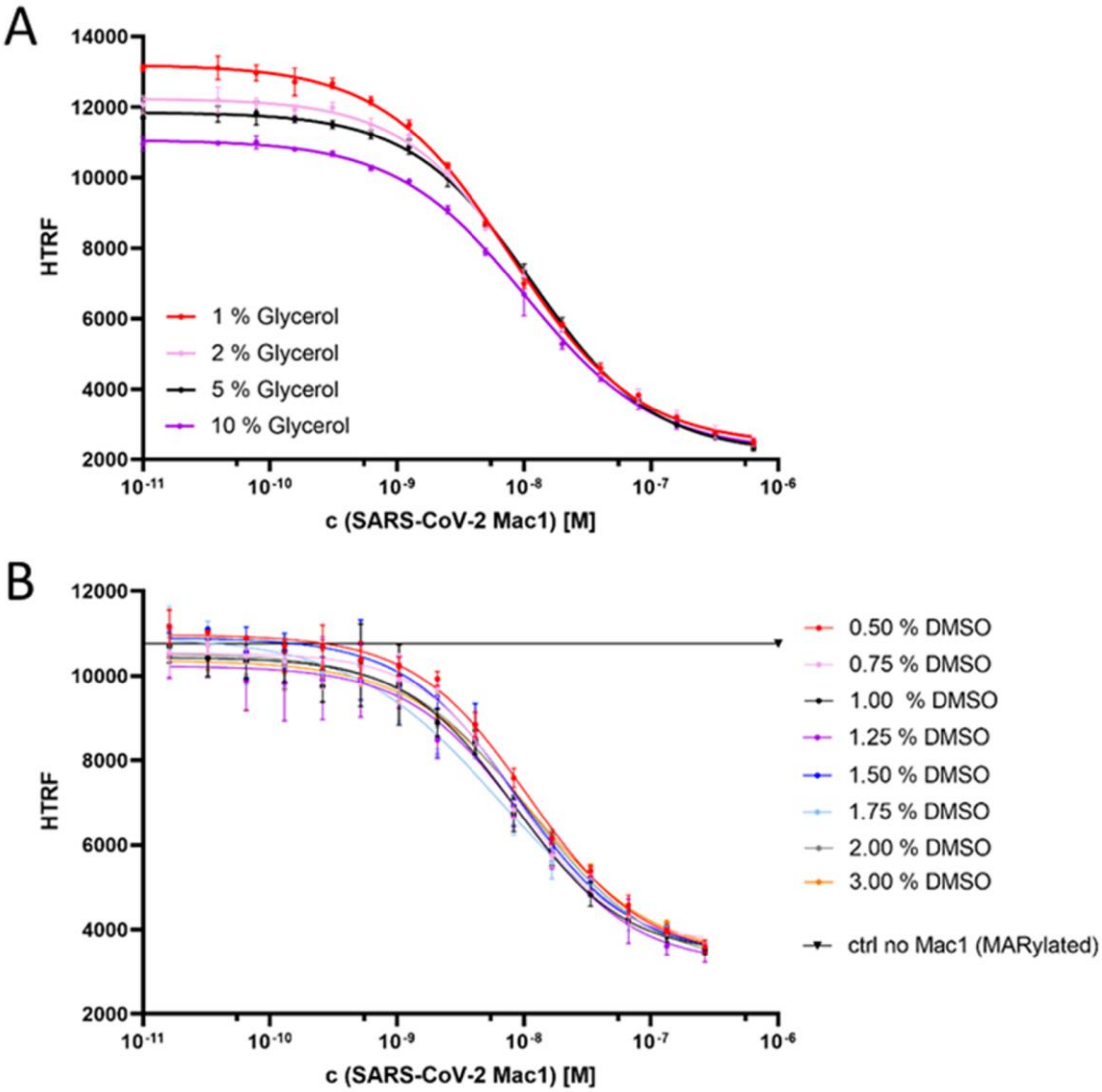

Figure S2: Evaluation of the influence of different glycerol and DMSO concentrations on the activity of SARS-CoV-2 Mac1, related to Figure 1.

(A) SARS-CoV-2 Mac1 was titrated from 0.04 up to 640 nM against 3 nM Tb-PARP10cat. The readout was performed with 200 nM sGFP-PARP14 Mac2/3 as described in Methods. This experiment was conducted in HRTF buffer containing either 1, 2, 5 or 10 % glycerol. A DMSO control (no Mac1) is the lowest concentration in each data set. Data represent the means ± SD of 3 technical replicates for each buffer condition.

(B) SARS-CoV-2 Mac1 was titrated from 0.0 up to 267 nM against 6 nM Tb-PARP10cat. The readout was performed with 200 nM sGFP-PARP14 Mac2/3 as described in Methods. This experiment was conducted in HRTF buffer containing either 0.5, 0.75, 1, 1.25, 1.5, 1.75, 2 or 3 % DMSO. A DMSO control (no Mac1) is the lowest concentration in each data set. Data represent the means ± SD of 3 technical replicates for each buffer condition.

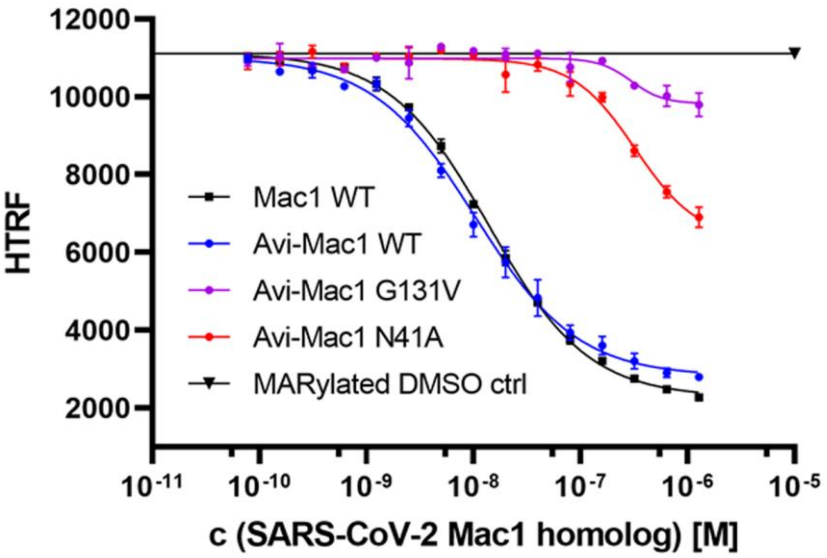

Figure S3: Comparison of the activity of SARS-CoV-2 Mac1 with two inactive mutants, related to Figure 1.

SARS-CoV-2 Mac1 homologs were titrated from 0.08 nM up to 1.28 µM against 3 nM Tb-PARP10cat. The readout was performed with 200 nM sGFP-PARP14 Mac2/3 as described in Methods. The term “Avi-” implies that the respective macrodomain features an N-terminal biotinylated Avi-tag. The G131V mutant is sterically inactive, while the N41A mutant is catalytically inactive. However, in the N41A mutant the ADPr binding pocket is not sterically blocked, and hence, can still accommodate ADPr or bind to MARylated substrate. Consequently, the N41A mutant competes with sGFP-PARP14 Mac2/3 for binding to Tb-PARP10cat, which explains the reduction in HTRF observed with this Mac1 mutant.

A DMSO control (without Mac1) is the lowest concentration in each data set. Data represent the means ± SD of 3 technical replicates each concentration tested.

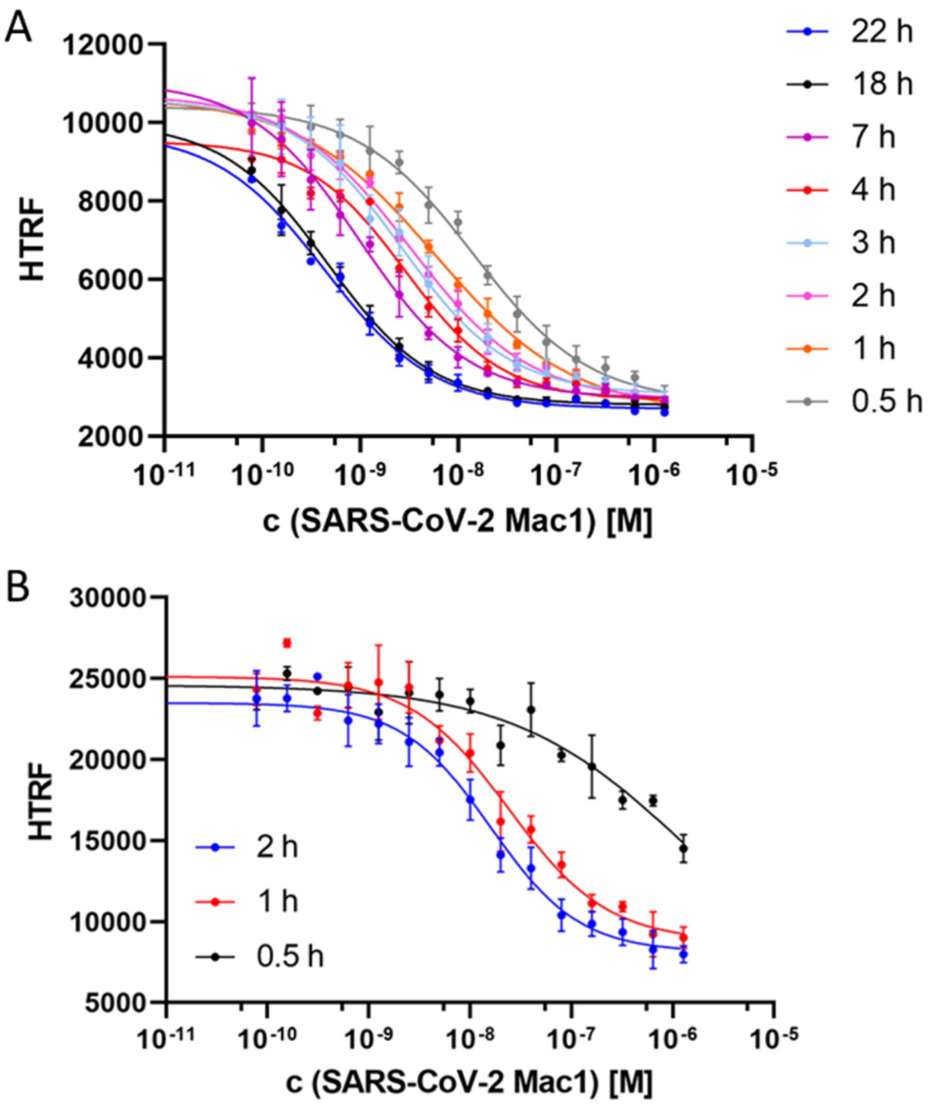

Figure S4: Evaluation of the influence of different incubation times on the deMARylation of SARS-CoV-2 Mac1 in the one-pot and in the wash setup, related to Table 1.

(A) SARS-CoV-2 Mac1 was titrated from 0.08 up to 640 nM against 3 nM Tb-PARP10cat in the one-pot setup. The readout was performed with 200 nM sGFP-PARP14 Mac2/3 after 0.5, 1, 2, 3, 4, 7, 18, 22 h as described in Methods. A negative control is the lowest concentration in each data set. Data represent the means ± SD of 3 technical replicates for each point of time.

(B) SARS-CoV-2 Mac1 was titrated from 0.08 up to 640 nM against 3 nM Tb-PARP10cat in the wash setup. The readout was performed with 200 nM sGFP-PARP14 Mac2/3 after 0.5, 1, 2 h as described in Methods. A negative control is the lowest concentration in each data set. Data represent the means ± SD of 3 technical replicates for each point of time.

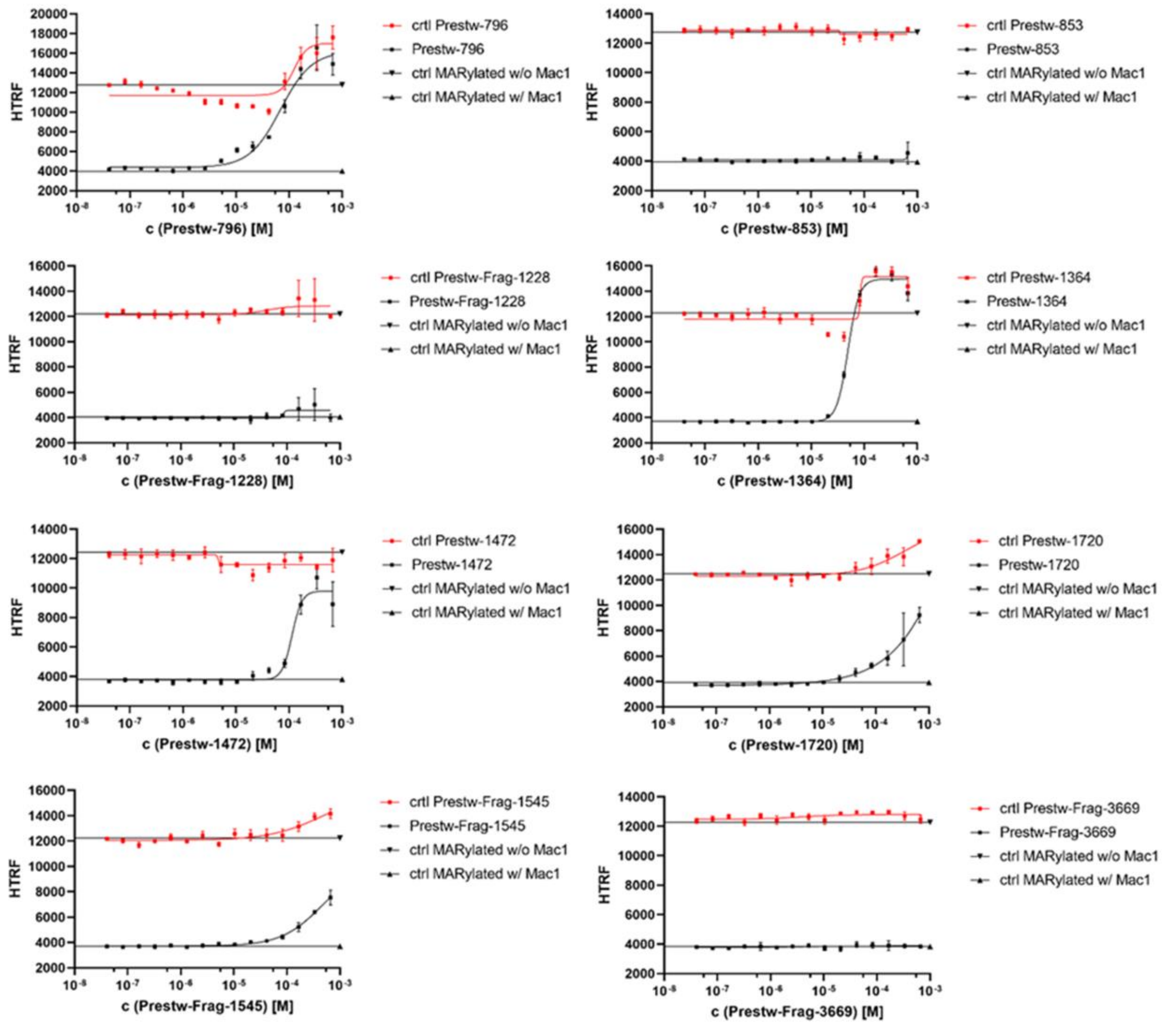

Figure S5: DeMAR assay (one-pot setup) dose-response experiments of the eight hits from the Prestwick SARS-CoV-2 Mac1 pilot screen, related to Figure 5.

(A) Each compound was titrated from 41 nM up to 667 µM added to 3 nM Tb-PARP10cat, and 80 nM SARS-CoV-2 Mac1 in the one-pot setup. The readout was performed with 200 nM sGFP-PARP14 Mac2/3 as described in Methods. A counter screen without Mac1 addition was also carried out for each compound titration (red). A DMSO control for each titration was performed. Data represent the means ± SD of 3 technical replicates for each concentration tested. Prestw-1472 is hexachlorophene (IC_{50}: 114 µM).

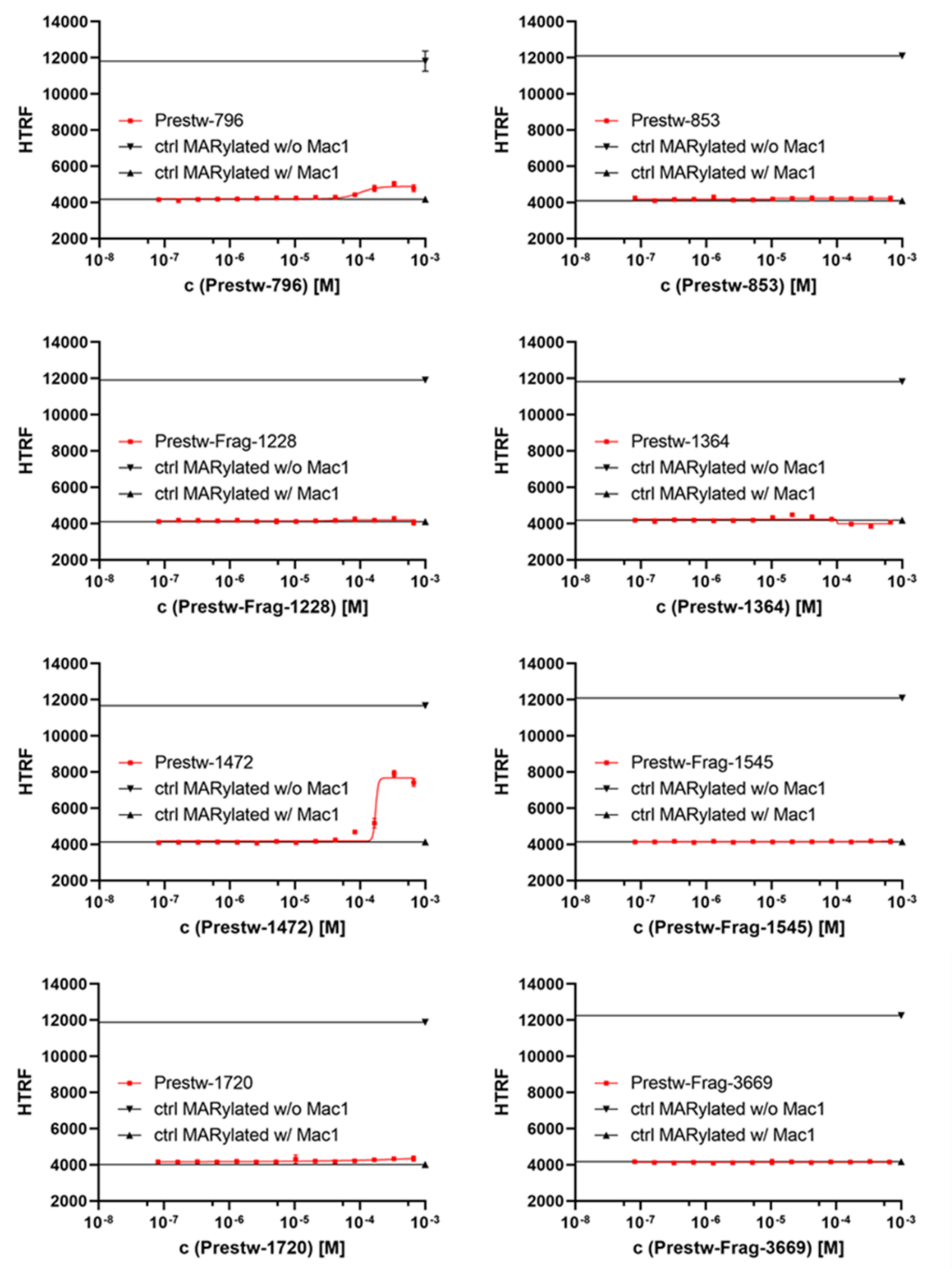

Figure S6: DeMAR assay (wash setup) dose-response experiments of the eight hits from the Prestwick SARS-CoV-2 Mac1 pilot screen, related to Figure 5.
Each compound was titrated from 82 nM up to 667 µM added to 6 nM Tb-ARTD10cat, and 80 nM SARS-CoV-2 Mac1 in the wash setup. The readout was performed with 200 nM sGFP-ARTD8 Mac2/3 as described in Methods. A DMSO control with and without Mac1 (3 technical replicates each) was performed to define the upper and lower assay plateau. Data represent the means ± SD of 3 technical replicates for each concentration tested. Prestw-1472 is hexachlorophene.

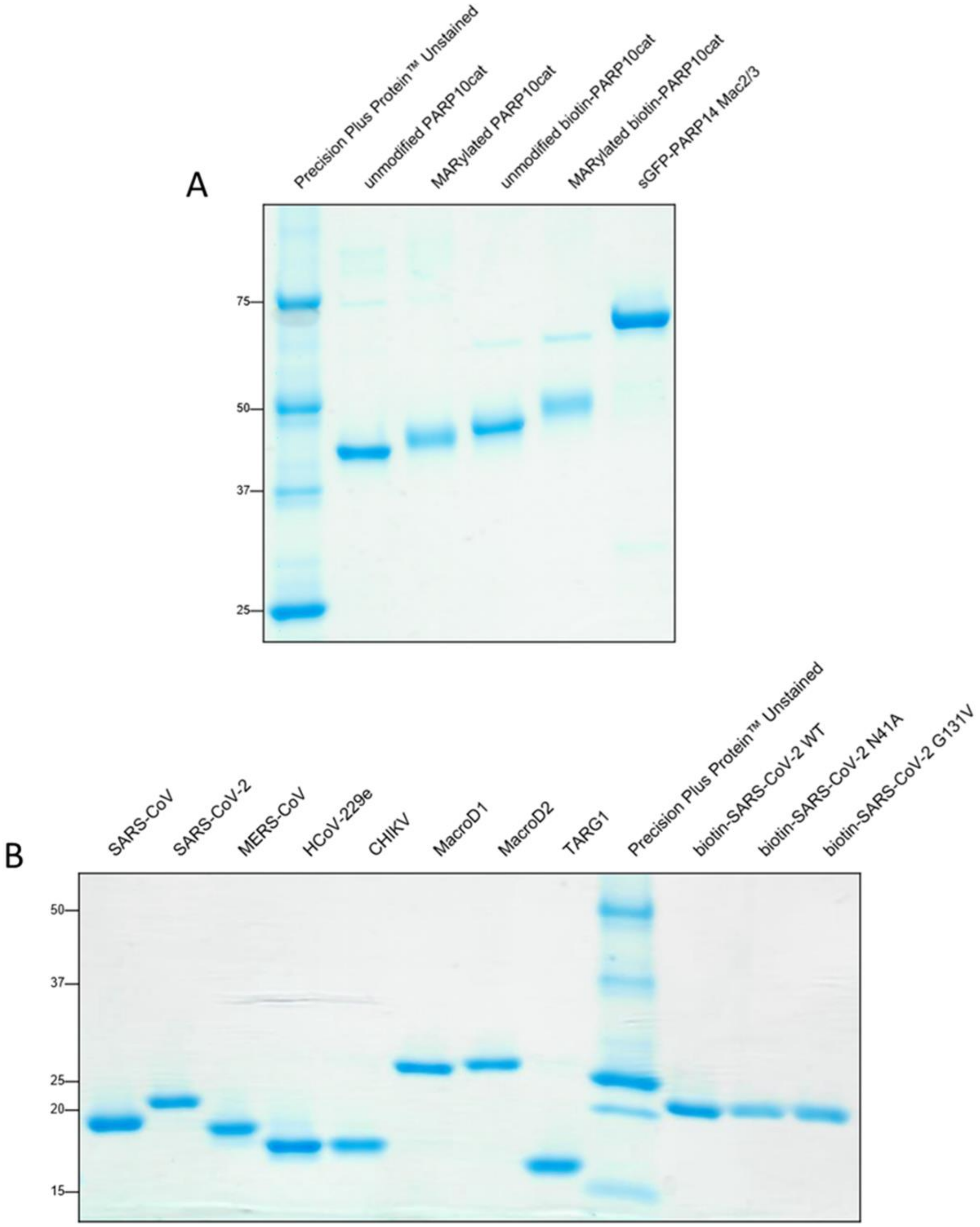

Figure S7: SDS-PAGE analysis of all of the protein preparations that were used for the DeMAR assay, related to Figure 1.
For SDS-PAGE, all protein samples were adjusted to a concentration of 0.05 mg/ml. Proteins were stained with Coomassie (Instant Blue™). (A) shows all PARP enzymes used in the study. (B) shows all Mac1 homologs used in the study.
